# ITGB4 as a novel serum diagnosis biomarker and potential therapeutic target for colorectal cancer

**DOI:** 10.1002/cam4.4216

**Published:** 2021-08-20

**Authors:** Xia Jiang, Jia Wang, Mengyu Wang, Mingda Xuan, Shuangshuang Han, Chao Li, Meng Li, Xiao‐Feng Sun, Weifang Yu, Zengren Zhao

**Affiliations:** ^1^ Hebei Key Laboratory of Colorectal Cancer Precision Diagnosis and Treatment The First Hospital of Hebei Medical University Shijiazhuang Hebei P.R. China; ^2^ Department of General Surgery The First Hospital of Hebei Medical University Shijiazhuang Hebei P.R. China; ^3^ Department of Internal Medicine The First Hospital of Hebei Medical University Shijiazhuang Hebei P.R. China; ^4^ Department of Endoscopy Center The First Hospital of Hebei Medical University Shijiazhuang Hebei P.R. China; ^5^ The First Department of Colorectal Surgery The Third Hospital of Hebei Medical University Shijiazhuang Hebei China; ^6^ Department of Oncology and Department of Biomedical and Clinical Sciences Linköping University Linköping Sweden

**Keywords:** colorectal cancer, CyTOF, diagnosis biomarker, ITGB4, Single‐cell level

## Abstract

**Purpose:**

To develop new and effective biomarkers for the diagnosis of colorectal cancer (CRC).

**Experimental design:**

The serum expression of ITGB4 (49 CRC and 367 HC) was detected by enzyme‐linked immunosorbent assay (ELISA), and its diagnostic value was analyzed using the receiver operating characteristic (ROC) curve. The sensitivity and specificity of ITGB4 in CRC diagnosis were calculated through statistical analysis. The optimal clinical cutoff value was calculated using the Youden index, and diagnostic efficacy was analyzed in a larger serum sample (98 CRC and 1631 non‐CRC). The expression of ITGB4 was measured by CyTOF (cell experimental technology) at the single‐cell level, and characteristics were analyzed using viSNE and SPADE TREE.

**Results:**

Serum ITGB4 and CEA levels were significantly higher in CRC patients than in HC and non‐CRC patients. The use of serum ITGB4 levels for the diagnosis of CRC has a high sensitivity (79%) but not high specificity when the clinical cutoff value was 0.70 ng/mL. However, the optimal cutoff value was 1.6 ng/mL with 86.2% specificity and 52.0% sensitivity, and the diagnostic efficacy was greatly improved with high specificity (82.0%) and sensitivity (71.4%) when combined with CEA. ITGB4 expression characteristics were measured and related to the expression of EpCAM, Ck8/18, and perforin at the single‐cell level. Single‐cell analysis showed that cell clusters with low expression of CK8/18 and ITGB4 were more sensitive to 5FU and radiotherapy (RT).

**Conclusions:**

ITGB4 is an effective diagnostic serum biomarker and a potential therapeutic target for CRC.

## Graphical abstract

The serum ITGB4 levels from CRC than in non‐CRC The best cutoff value of ITGB4 was defined as 1.6 ng/mL and combined with CEA, the diagnostic efficiency is the best ITGB4 is a potential serum biomarker for the diagnosis of CRC.

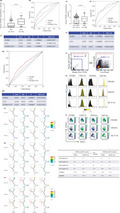

**Highlight**

Serum ITGB4 levels were higher in colorectal cancer (CRC) patients than in non‐CRC patients.

The best cutoff value of ITGB4 was 1.6 ng/mL and this had the highest diagnostic efficacy in combination with CEA.

ITGB4 is a potential serum biomarker for CRC.

## 1 INTRODUCTION

Colorectal cancer (CRC) is the third most common malignancy and is associated with increased morbidity and mortality.^1^ The 5‐year survival rate for patients with early CRC was 90%, while that for patients with advanced CRC was only 14%.^2^, ^3^ Therefore, early accurate diagnosis is of great significance in improving the survival of patients with CRC. Serum carcinoembryonic antigen (CEA), as a noninvasive serum biomarker, has good specificity for identifying occult CRC, but its application is limited owing to its low sensitivity of only 40%–60%.^4^, ^5^ CEA is used in the clinic to monitor CRC, and it is well established that it is not a good screening method of CRC patients. Fecal occult blood tests, especially the fecal immunochemical tests (FITs), have been fully implemented in most countries for almost a decade as a valid non‐invasive CRC screening method with 79% sensitivity and 94% specificity.^6^ Colonoscopy is usually regarded as the gold standard for early detection of CRC; however, FIT and colonoscopy are still limited as an effective approach for early detection owing to low patient compliance, only about 50% and 44% in China.^7^ Thus, it is necessary to develop a new serum biomarker with high sensitivity and non‐invasiveness for the detection of CRC.

The integrin (ITG) molecule is a cell‐surface receptor that is responsible for extracellular matrix interactions^8^, ^9^ and is formed by the noncovalent associations of α and β dimers. To date, 18 α and 8 β subunits have been found to form a number of distinct integrins.^10^ ITGs are involved in the regulation of a variety of cell signaling pathways, including migration, invasion, differentiation, proliferation, and survival.^11^, ^12^, ^13^, ^14^ Among them, ITGB4, located at 17q25.1, has been reported to be aberrantly expressed in several cancers, including breast, pancreatic, lung, and gastric cancers, and may be positively associated with poor prognosis.^15^, ^16^, ^17^, ^18^, ^19^

Previous studies have revealed abnormally high ITGB4 expression in CRC tissue, and ITGB4 was confirmed to be associated with a prognostic factor of CRC.^8^ However, the effectiveness of ITGB4 in the diagnosis of CRC requires further verification. In the present study, we studied the biological function of ITGB4 in human CRC cells and focused on verifying the accuracy and application of ITGB4 in the serum diagnosis of CRC. These findings indicate that serum ITGB4 is a novel potential diagnostic biomarker for CRC.

## 2 MATERIALS AND METHODS

### 2.1 Participants, sample collection, and ethical approval

Informed consents were obtained from all participants. The study was approved by the Clinical Research Ethics Committee of the First Affiliated Hospital of Hebei Medical University (No. 2013106 & 201707007).

This serological study was divided into two parts, as shown in Figure S1. In part I (2015–2017), to analyze the diagnostic value and clinical serum cutoff value of ITGB4, 417 participants undergoing colonoscopy, including 50 CRC and 367 healthy controls (HCs), were retrospectively analyzed for the serum concentration of ITGB4 and CEA by ELISA. One CRC case was rejected because of hemolysis. The ethical approval number was 2013106 approved by the Clinical Research Ethics Committee of the First Affiliated Hospital of Hebei Medical University. The clinical characteristics of the 416 included participants are presented in Table S1. In part II (Jan 2018–Nov 2020), a prospective nested case–control study was conducted with 1729 participants, all participants signed the informed consent and contributed serum to determine the concentration of ITGB4 and CEA through ELISA, and then underwent colonoscopy, including 98 CRC and 1631 non‐CRC participants; the CRC diagnostic efficacy of ITGB4 at a clinical cutoff value was evaluated. The best cutoff values were selected as determined using the Youden index method.^20^ The ethical approval number was 201707007 approved by the Clinical Research Ethics Committee of the First Affiliated Hospital of Hebei Medical University. The clinical characteristics of the 1729 participants are listed in Table S2. According to the colonoscopy biopsy pathological diagnosis, among 1631 non‐CRC participants, 532 were colorectal adenoma (CRA) patients and 1099 were HCs (Table S2). Participants were diagnosed by colonoscopy and biopsy histopathology.

Between 2015 and 2020, blood samples were collected into 5‐ml evacuated tubes and coagulating tubes (VACUETTE^®^, Greiner Bio‐One). To isolate serum, 5 ml of blood was centrifuged at 3,000 rpm for 15 min at 4°C. The upper serum was collected and stored at −80°C in the clinical biobank of our hospital until use.^21^

### 2.2 Serological detection of ITGB4 by ELISA

The ELISA kit (Biorbyt) was used to detect ITGB4 levels in serum samples. Serum was assayed using the Human ITGB4 ELISA kit (Biorbyt) according to the manufacturer's instructions. Serum samples (100 μL) and diluted standards were added to 96‐well ELISA plates, which were pre‐coated with an antibody specific to ITGB4. After incubation at 37°C for 2 h, the liquid was removed. Then, 100 μL of biotin‐conjugated ITGB4‐specific antibody at working concentration was added to each well and incubated for 1 h at 37°C. After the plates were washed three times, 100 μL of avidin conjugated to horseradish peroxidase (HRP) was added to each well and incubated for 1 h at 37°C. The wash process was conducted for a total of five times, followed by the addition of 90 μL of TMB substrate solution to each well, incubation at 37°C for 15–25 min without light, and addition of 50 μL of stop solution to each well. Measurements were taken at 450 nm immediately using a microplate reader (TECAN).

### 2.3 Serological detection of CEA by chemiluminescence assay

CEA concentration was quantitatively measured using chemiluminescence immunoassay assay kits (Beckman Coulter), according to the manufacturer's instructions. Normal reference values for CEA were assumed to be 0–5 ng/mL.

### 2.4 Cell culture

The human CRC cell lines HCT116, SW480, and SW620 were purchased from ATCC. HCT116 cells were cultured in McCoy's 5A Medium (Gibco) supplemented with 10% fetal bovine serum (FBS; Gibco). SW480 and SW620 cells were cultured in DMEM (Gibco) supplemented with 10% FBS. Cultured cells were maintained in a humidified 5% CO_2_ atmosphere at 37°C.^22^, ^23^

### 2.5 Radiation

Cells were irradiated with 2.5 Gy single doses, and the field size at SSD was 30 × 30 cm. Single‐cell protein expression was measured using CyTOF mass cytometry (CyTOF, Fluidigm Corporation).

### 2.6 Chemotherapy

Cells were treated with 5‐fluorouracil (5‐FU, 1 μg/mL) and the single‐cell protein expression was detected with CyTOF after 48 h of treatment.^24^

### 2.7 Mass cytometry

As reported previously,^25^ the single‐cell protein expression of three million cells was measured by CyTOF2. After methanol fixation (30 min, −20°C), the staining solution was prepared (83 µL staining buffer and 1 µL antibody) and the cells were stained for 30 min at room temperature. The panel is presented in Table S3. After washing with PBS, single‐cell data were collected in FCS files. CyTOF data were normalized using a MATLAB‐based software program called bead normalization.^25^ Normalized data were gated using the Cytobank website to generate clean data by eliminating beads and cisplatin‐positive events. Gating data were analyzed using Vortex and Cytobank software.

### 2.8 viSNE

viSNE, a mapping technique for transforming high‐dimensional cytometry data into two‐dimensional visualizations, was performed using Cytobank. Normalized single‐cell data were distributed using the Barnes–Hut implementation of the t‐SNE algorithm.^26^ Single‐cell data of cell lines were visualized; a point in the viSNE plot represented one cell, and the expression level of each protein was visualized as heat intensity on the viSNE map by global single‐cell view.

### 2.9 SPADE analysis

The cell events were clustered using Vortex, a graphical tool for cluster analysis of single‐cell data. Divisive marker trees (DMT), a form of SPADE analysis, were laid out according to the developer instructions (https://github.com/nolanlab/vortex/wiki/Getting‐Started). The following settings were implemented: arcsinh cofactor = 5, target number of clusters = 150, and downsample to target number of events = 5.

### 2.10 RNA extraction and real‐time quantitative polymerase chain reaction (qRT‐PCR)

Total RNA was extracted from transfected cells using TRIzol Reagent (Invitrogen). To quantify ITGB4, cDNA was reverse transcribed using PrimeScript™ RT reagent kit (Takara).^27^ qRT‐PCR was performed using SYBR^®^ Green Master Mix (Vazyme). GAPDH was used as an internal control for mRNA. The primer sequences were as follows: ITGB4 (forward), 5‐TCTCTCAGAGTGAGCTGGCAG‐3; ITGB4 (reverse): 5‐TTCAGCAGCTGGTACTCCAC‐3; β‐actin (forward): 5‐CAGCCATGTACGTTGCTATCCAGG‐3; β‐actin (reverse) 5‐AGGTCCAGACGCAGGATGGCATG −3. All experiments were performed in triplicate on an ABI7500 Sequence Detection System, and mean cycle threshold (CT) data were obtained. The relative amount normalized to the internal control was calculated using the equation 2‐^ΔΔCT^.

### 2.11 Cell transfection

siRNA‐ITGB4 (Genepharma) and a negative control (Gibco) were used for cell transfection using Lipofectamine 2000 (Invitrogen). Transfected cells were harvested 48 h after transfection. Transfection efficiency was verified by qRT‐PCR.

### 2.12 Cell proliferation assay

The transfected cells were plated at a density of 2500 cells/well in 96‐well plates and incubated overnight in medium supplemented with 10% FBS. Cell proliferation was measured using a Cell Counting Kit‐8 (Dojindo) at 24, 48, 72, and 96 h post‐transfection, following the manufacturer's instructions. The absorbance at 450 nm was measured using a Promega GloMax luminescence detector (Promega). The experiment was performed in triplicate.

### 2.13 Wound healing assay

When the transfected cells in the 6‐well plates reached a growth density of 85%, the confluent monolayers were scratched with a pipette tip to create a gap to simulate a wound and the non‐viable cells were washed with PBS. The transfected cells were cultured in McCoy's 5A medium for HCT116 or DMEM for SW480. Images of the plates were obtained under a microscope (Nikon) at 0, 24, and 48 h.^28^

### 2.14 Migration assay

Transwell assays were performed in uncoated pore chambers for migration. To measure migration, 3.5 × 10^4^ transfected cells were resuspended in 300 µL of serum‐free McCoy's 5A medium for HCT116 or DMEM for SW480 and added to the upper chamber, whereas 800 µL of McCoy's 5A medium for HCT116 or DMEM for SW480 containing 10% FBS was added to the lower chamber. After 24 h of incubation, the cells on the upper surface of the membrane were gently removed using cotton swabs, and the cells were stained using a Diff‐Quick stain kit according to the manufacturer's protocol and counted blindly (five random fields per chamber).

### 2.15 Statistical analysis

All statistical analyses were performed using SPSS version 21.0 software (IBM SPSS Inc.). Data are presented as mean ± standard deviation for normally distributed continuous data, as median (interquartile range, Q25–Q75) for abnormally distributed continuous data, or as actual values for categorical data. Baseline characteristics were summarized using descriptive statistics. Groups were compared using *χ* ^2^ tests for categorical variables, Mann–Whitney U tests for continuous variables, and one‐way ANOVA tests for three or more independent groups. Receiver operating characteristic (ROC) curves were used to compare the diagnostic performance of each biomarker. The predicted probability value was calculated using binary logistics regression, and used to draw ROC curve of combination ITGB4 and CEA. The area under the ROC curve (AUC) of each biomarker for distinguishing CRC and non‐CRC patients, as well as the optimal cutoff value, sensitivity, specificity, and 95% confidence interval (95% CI) were calculated using IBM SPSS 21.0. Statistical significance was set at *p* < 0.05.

## 3 RESULTS

### 3.1 ITGB4 was evaluated as a potential serum diagnostic marker for CRC

ITGB4 was evaluated as a potential diagnostic marker for CRC through the cross‐analysis of multiple databases. First, the expression characteristics and location of all proteins were analyzed using the compartment database (https://compartments.jensenlab.org/). In total, 2694 proteins expressed in EVs and exosomes were screened. Next, the results of the GSE1133 dataset were visualized using BIOGPS (http://biogps.org/) and GEO2R (https://www.ncbi.nlm.nih.gov/geo/geo2r/?acc=GSE1133) websites, and the expression of 2024 proteins was compared. Thirty‐four genes (Table S4) were identified through the following two schemes: (a) the expression level in colorectal adenocarcinoma (COAD) was the highest and was higher than that in colon tissue (fold change ≥ 5, including 22 proteins) or (b) the expression level in COAD was higher than that in colon tissue (fold change ≥ 30, including 12 proteins). Five proteins (ITGB4, INHBB, TNFRSF6B, CTDSPL, and KRT18) were randomly selected for tissue expression analysis (n = 50). The expression of ITGB4 was increased only in cancer tissues. In previous studies, we had additionally analyzed ITGB4 expression in CRC tissues and adjacent normal tissues and had confirmed that ITGB4 was highly expressed in CRC tissues.^8^

**FIGURE 1:**
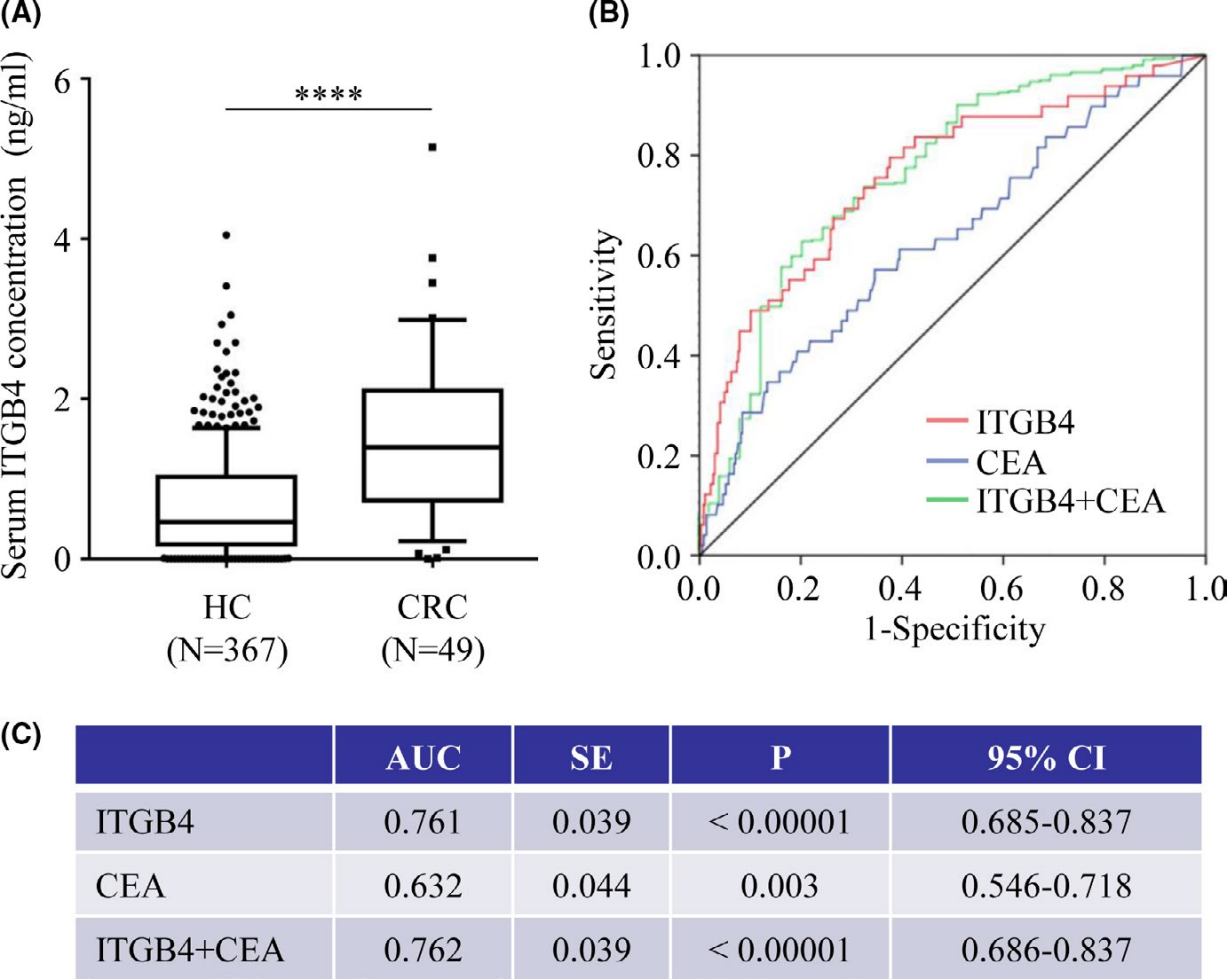
Serum concentration of ITGB4 and its ability to distinguish CRC patients (N = 49) from HCs (N = 367) in a small‐sample retrospective study. (A) Serum concentration of ITGB4 in 416 serum samples (49 CRC and 367 HCs). (B) ROC curve of ITGB4 (red), CEA (blue), and ITGB4 combined with CEA (green). (C) AUC and *p* value of ITGB4, CEA, and ITGB4 combined with CEA

In part I, the serum concentrations of ITGB4 in 416 serum samples (49 CRC patients and 367 HCs) were analyzed by ELISA. The median serum ITGB4 concentration in the CRC samples was 1.396 ng/mL (Q25–Q75: 0.7327–2.104 ng/mL), which was significantly higher than that in HCs (0.4629 ng/mL, Q25–Q75: 0.182–1.026 ng/mL, *p* < 0.05) (Figure 1A). ITGB4 showed effectiveness in discriminating CRC patients from HCs by the ROC curve, with an AUC of 0.761 (95% confidence interval: 0.685–0.837; *p* < 0.0001; Figure 1B,C). At the best cutoff value (0.70 ng/mL), which maximized the sum of sensitivity and specificity by ROC analysis, ITGB4 could discriminate CRC patients from HCs with a sensitivity of 79.59% and specificity of 62.4% in part I, which included 49 CRC patients and 367 HCs (Figure 1B,C). The AUC of CEA was 0.632 (95% confidence interval: 0.546–0.718; *p* = 0.003), and CEA could discriminate CRC patients from HCs with a sensitivity of 16.3% and specificity of 93.2% at a cutoff value of 5 ng/ml (Figure 1B,C). Compared with diagnosis with only ITGB4, combined diagnosis with ITGB4 and CEA did not significantly increase the AUC of 0.762 (95% confidence interval: 0.686–0.837; *p* < 0.0001; Figure 1B,C).

### 3.2 Diagnostic efficacy of the clinical cutoff value of ITGB4 in discriminating CRC patients from non‐CRC participants

A prospective real‐world nested case–control study was conducted from January 2018 to November 2020 in our hospital to further analyze the applicability of the ITGB4 clinical cutoff value (0.70 ng/mL). A total of 1729 volunteers participated in this prospective study. Among them, 98 patients were diagnosed to have CRC by colonoscopy biopsy pathology, and the remaining 1631 were diagnosed as non‐CRC. Among 1631 non‐CRC participants, 532 were diagnosed to have colorectal adenomas (CRAs) by colonoscopy biopsy pathology, and 1099 participants were HCs confirmed by colonoscopy.

**FIGURE 2:**
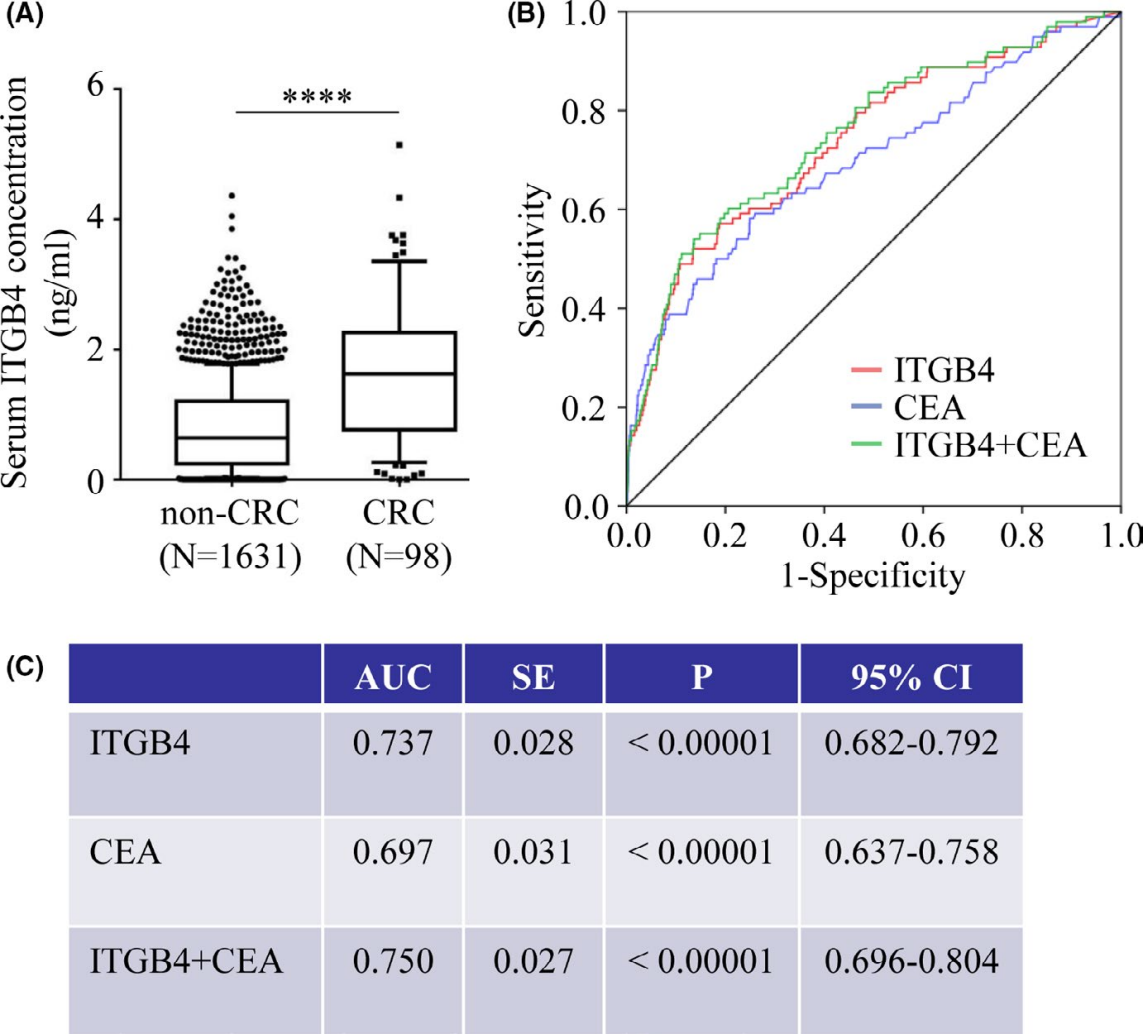
Serum concentration of ITGB4 and its ability to distinguish CRC patients (N = 98) from non‐CRC participants (N = 1631) in a large‐sample prospective real‐world nested case–control study. (A) Serum concentration of ITGB4 in 98 CRC patients and 1631 non‐CRC participants. (B) ROC curve of ITGB4 (red), CEA (blue), and ITGB4 combined with CEA (green). (C) AUC and *p* value of ITGB4, CEA, and ITGB4 combined with CEA

As shown in Figure 2A, the serum ITGB4 concentration of 1.624 ng/mL in CRC patients (Q25–Q75: 0.763–2.261 ng/mL) was significantly higher than that in non‐CRC participants (0.638 ng/mL, Q25–Q75: 0.246–1.206 ng/mL, *p* < 0.05). First, the diagnostic efficacy of ITGB4 in discriminating CRC patients from non‐CRC participants was analyzed. ITGB4 still showed better performance than CEA in distinguishing CRC patients from non‐CRC participants. The AUC of ITGB4 was 0.737 (95% confidence interval: 0.682–0.792; *p* < 0.0001; Figure 2A,B) with a sensitivity and specificity of 79.6% and 53.2%, respectively (Figure 2A,B). The AUC of CEA was 0.697 (95% confidence interval: 0.637–0.758; *p* < 0.0001); CEA at a cutoff value of 5 ng/ml could discriminate CRC patients from HCs with a sensitivity of 32.7% and specificity of 95.2% (Figure 2A,B). We also evaluated the utility of combining ITGB4 and CEA for the diagnosis of CRC and found that this combination resulted in an increased AUC (0.750) compared with the AUC of only ITGB4 (0.737) or CEA (0.697).

The diagnostic efficacy of the clinical cutoff value for ITGB4 in discriminating CRC patients from non‐CRC participants was further analyzed. As shown in Table 1, when 0.70 ng/mL was used as the cutoff value of ITGB4 for CRC diagnosis and only ITGB4 was used as a biomarker, 78 CRC patients were correctly diagnosed, 20 CRC patients were missed, and 764 of 1631 non‐CRC participants were misdiagnosed as CRC (false positives); thus, the misdiagnosis rate was obviously high. With the same cutoff value for ITGB4, when the combination of ITGB4 and CEA was used as a biomarker in the diagnosis of CRC, only 11 of 98 CRC patients were missed, but 802 of 1631 non‐CRC participants were misdiagnosed as CRC (false positives). The use of 0.70 ng/mL as the clinical cutoff value of ITGB4 for CRC diagnosis was highly sensitive but not specific.

**TABLE 1:** Performance of ITGB4 or CEA alone and in combination with each other for colorectal cancer diagnosis

	ITGB4 (cutoff = 0.7 ng/mL)		CEA (5 ng/mL)	ITGB4 (cutoff = 1.6 ng/mL)	
	ITGB4	ITGB4 + CEA		ITGB4	ITGB4 + CEA
True positive (N)	78	87	32	51	70
False positive (N)	764	802	78	225	294
False negative (N)	20	11	66	47	28
True negative (N)	867	829	1553	1406	1337
Sensitivity	79.6%	88.8%	32.7%	52.0%	71.4%
Specificity	53.2%	50.8%	95.2%	86.2%	82.0%

To find a better clinical cutoff value, the Youden index was calculated using the data from the prospective study (part II). The best cutoff value of 1.6 ng/mL of ITGB4 could discriminate CRC patients from non‐CRC participants with a sensitivity of 52.0% (51 true‐positive cases and 47 false‐negative cases) and specificity of 86.2% (225 false‐positive cases and 1406 true‐negative cases) in part II (Table 1). Combining ITGB4 (1.6 ng/mL) with CEA in the diagnosis of CRC had high specificity (82.0%; 294 false‐positive cases and 1337 true‐negative cases) and an improved sensitivity of 71.4% (70 true‐positive cases, 28 false‐negative cases, Table 1). Furthermore, the efficacy of the two clinical cutoff values for distinguishing CRC patients (N = 98) from HCs (N = 1099) was analyzed. Each clinical cutoff value of ITGB4 showed improved diagnostic power in distinguishing CRC patients from HCs, with a larger AUC (Figure S2A,B) and higher specificity (Table S5).

### 3.3 Diagnostic efficacy of ITGB4 in discriminating CRA patients from HC participants

**FIGURE 3:**
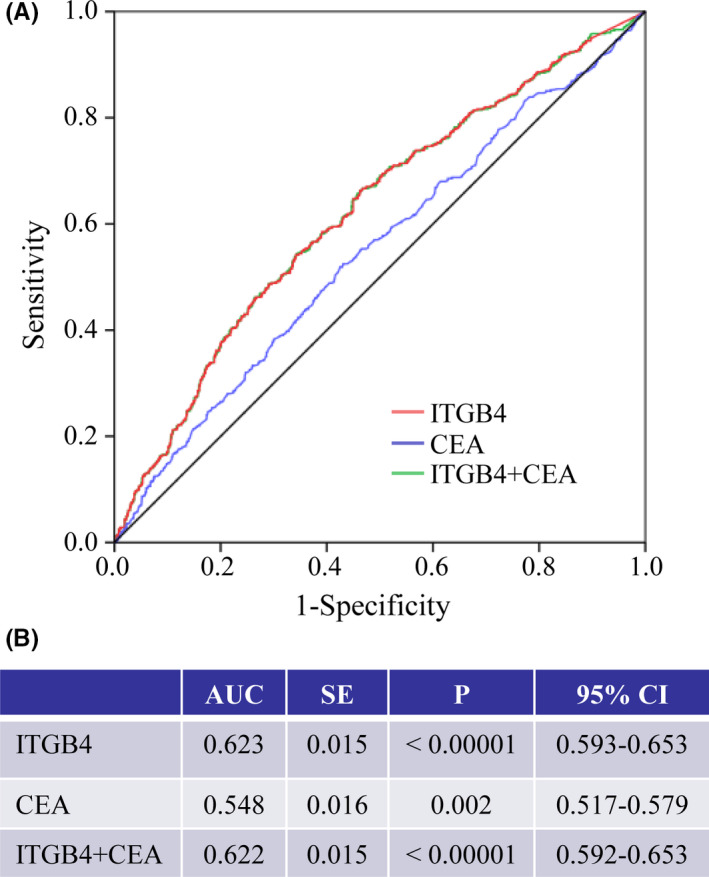
Diagnostic efficacy of ITGB4 in discriminating CRA patients (N = 532) from HCs (N = 1099). (A) ROC curve of ITGB4 (red), CEA (blue), and ITGB4 combined CEA (green). (B) AUC and *p* value of ITGB4, CEA, and ITGB4 combined with CEA

The 1631 non‐CRC participants included 532 CRA patients and 1099 HCs. We analyzed the effectiveness of ITGB4 in distinguishing CRA patients from HCs. The median ITGB4 concentration in CRA patients was 0.892 ng/mL (Q25–Q75: 0.363–1.418 ng/mL), which was higher than that in HCs (median: 0.507 ng/mL, Q25–Q75: 0.199–1.065 ng/mL) (Figure S3). For an ITGB4 cutoff value of 0.70 ng/mL, the AUC of ITGB4 was only 0.623 (Figure 3), with 58.52% sensitivity and 60.88% specificity. Combining ITGB4 with CEA did not improve the diagnostic efficacy (Figure 3).

### 3.4 ITGB4 is a potential therapeutic target for CRC

Previous research has found ITGB4 to be an independent predictor of survival and to be expressed in cells resembling CRC tumor‐budding cells.^8^, ^25^ Our results confirmed that ITGB4 expression in serum (median: 1.834 ng/mL, Q25–Q75: 0.904–2.573 ng/mL, *p* = 0.038, Figure S4A) and tissues (median: 0.019 fold, Q25–Q75: 0.013–0.032 fold, *p* < 0.0001; Figure S4B) of metastatic CRC patients (N = 41) was higher than that in serum (median: 1.408 ng/mL, Q25–Q75: 0.707–2.080 ng/mL, Figure S4A) and tissues (median: 0.009 fold, Q25–Q75: 0.006–0.017 fold, Figure S4B) of non‐metastatic CRC patients (N = 55). It has been reported that ITGB4 is associated with CRC metastasis.^29^ We also confirmed that knockdown of ITGB4 by si‐RNA (Figure 5) reduces the metastasis of HCT116 (Figure S6) and SW480 cells (Figure S7).

**FIGURE 4:**
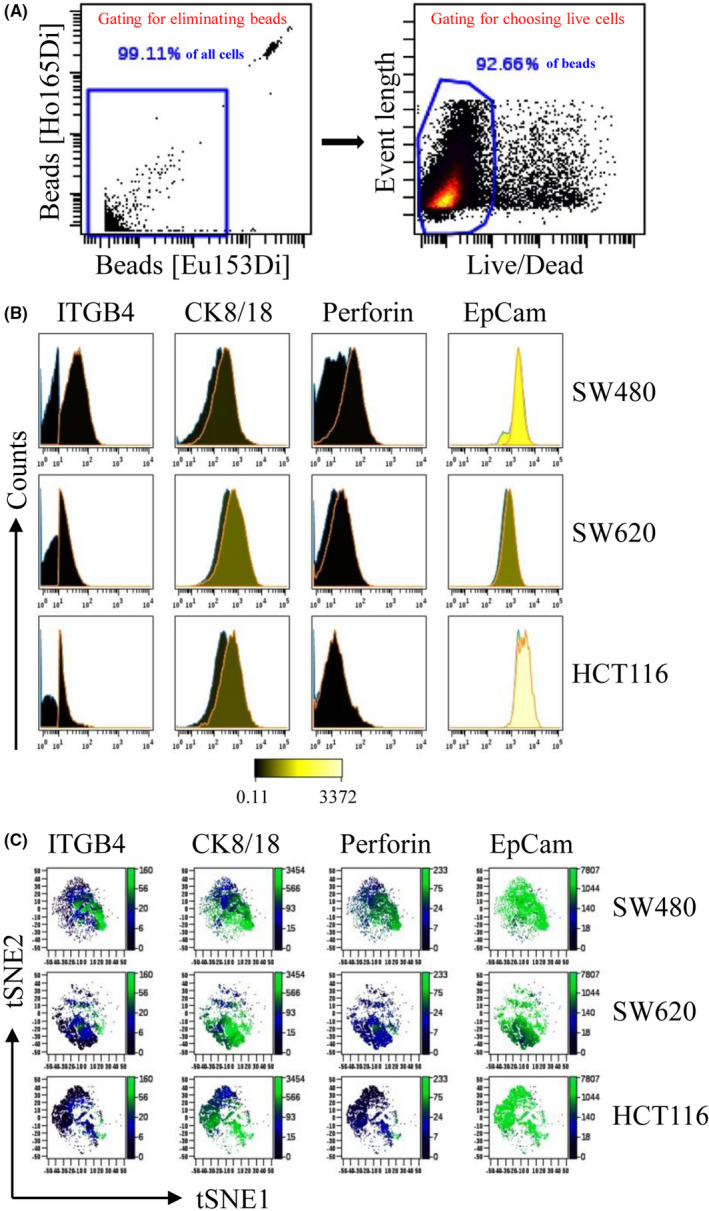
Expression characteristics of ITGB4 at the single‐cell level using live cells. (A) The gates eliminating beads and dead cells. (B) The histograms of ITGB4, CK8/18, EpCAM, and perforin expression in the ITGB4+ (orange line) and ITGB4‐ (blue line) cells. Change in color from black to yellow indicates the protein expression level from low to high. (C) viSNE diagrams depicting ITGB4, CK8/18, EpCAM, and perforin expression of live cells. A dot represented a single cell, and the change in color from black to green indicates low to high expression

To analyze the expression characteristics of ITGB4, single‐cell level protein expression was measured by CyTOF. Beads and dead cells were eliminated by gating (Figure 4A). The cells were grouped into two groups by ITGB4 expression: ITGB4^+^ and ITGB4^−^ groups (Figure 4B) in HCT116, SW480, and SW620 cells. The expression level of CK8/18 was higher in the ITGB4^+^ group than in the ITGB4^−^ group (Figure 4B) in SW480, SW620, and HCT116 cells. The expression level of perforin was higher in the ITGB4^+^ group than in the ITGB4^−^ group (Figure 4B) in SW480 and SW620 cells. The EpCAM expression was higher in the ITGB4^+^ group than in the ITGB4^−^ group (Figure 4B) in SW480 and SW620 cells. Single‐cell cluster analysis by viSNE also showed that the high expression of ITGB4 was related to CK8/18, EpCAM, and perforin expression (Figure 4C). Interestingly, the result of viSNE showed that the ITGB4 expression consistently had the best correlation with the expression of perforin in the three cell lines (Figure 4C).

**FIGURE 5:**
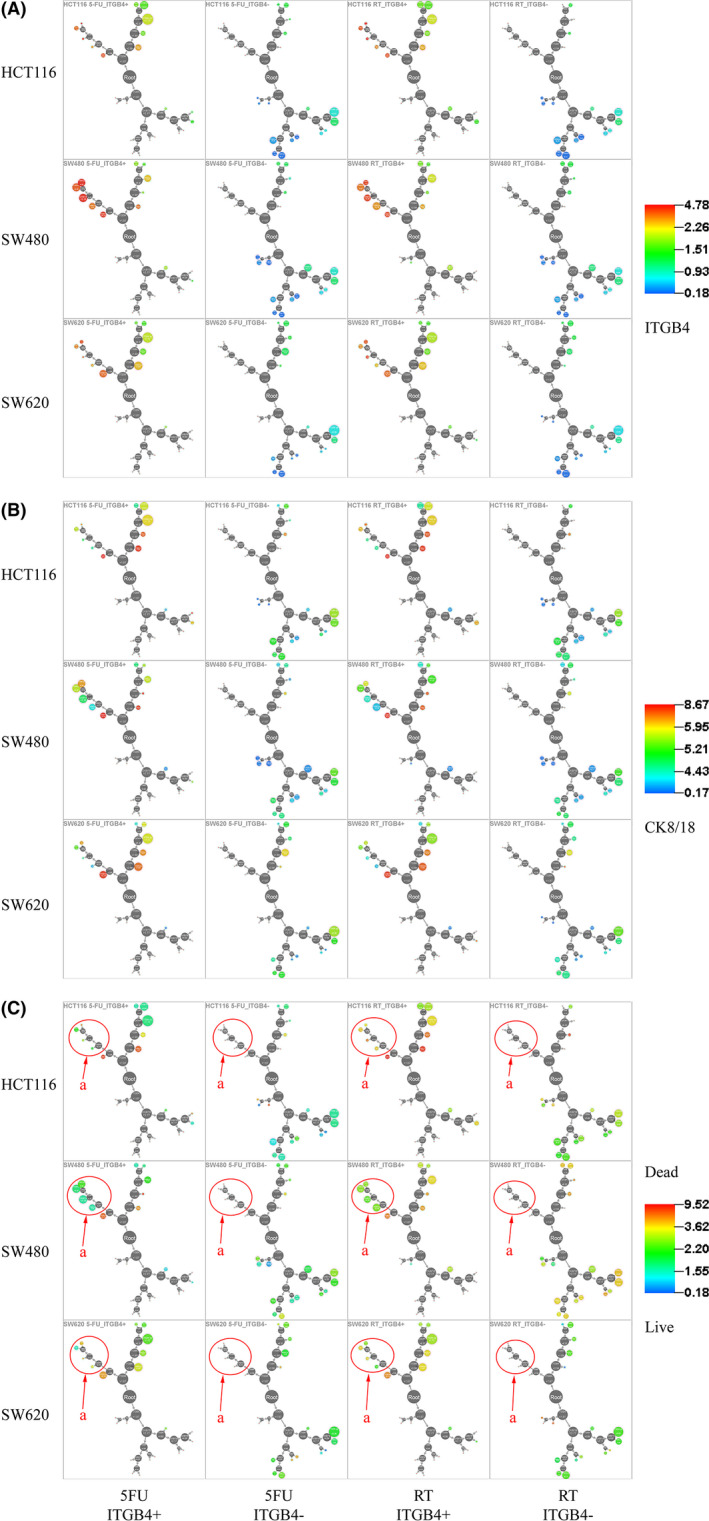
The contrast SPADE tree of ITGB4+ and ITGB4‐ cells with 48 h of treatment with 5‐FU or RT. (A) ITGB4 expression in HCT116, SW480, and SW620 cells with 5‐FU or RT treatment (only using the beads gate) depicted in the SPADE tree. One colorful node represents one cluster of cells, and the change in color from blue to red indicates low to high expression. (B) CK8/18 expression in HCT116, SW480, and SW620 cells with 5‐FU or RT treatment (only using the beads gate) depicted in the SPADE tree. One colorful node represents one cluster of cells, and the change in color from blue to red indicates low to high expression. (C) Live or dead HCT116, SW480, and SW620 cells are depicted in the SPADE tree after 5‐FU or RT treatment (only using the beads gate). One colorful node represents one cluster cells, and the change in color from blue to red indicates proportion of live to dead cell (blue indicates live cells and red indicates dead cells). Arrows a and b point to two clusters with high mortality; these two clusters with high mortality only appeared in ITGB4+ cells

After treated with 5‐FU (1 μg/mL) and RT (2.5 Gy) alone for 48 hours, a SPADE tree (DMT type) was used to perform cell cluster analysis for single‐cell protein expression. The results showed that the main determinants of clustering were the expression of ITGB4 and CK8/18. The SPADE tree results of ITGB4 (Figure 5A) expression, CK8/18 (Figure 5B) expression, and cell survival or death (Figure 5C, using Pt195Di channel) were presented one by one. The cluster characteristics are shown in Figure S8. In ITGB4^+^ group, there are less dead cells in zone "a", which means that the cells distributed in region "a" were more resistant to 5‐FU and radiotherapy (RT). Cells in a region with ITGB4 expression levels between 2.58 and 10 and CK8/18 expression levels less than 5.74 were more sensitive to 5‐FU and RT in ITGB4^−^ group (Figure 5C). This result showed that ITGB4 expression may be related to the sensitivity of the cells to chemoradiotherapy.

## 4 DISCUSSION

Globally, CRC is the third most common cancer and the second most lethal cancer. The 5‐year survival rate of most CRC cases can be significantly improved if detected sufficiently early. Therefore, the identification of serum biomarkers for early CRC is critical for the early diagnosis, treatment, and improvement of clinical outcomes of CRC patients. CEA is the most common clinical serum biomarker for CRC and is correlated with tumor stage, prognosis, and scope of surgical resection of individual patients. Although the current application of CEA has high specificity, its low sensitivity limits its use, particularly in large‐scale screening and early diagnosis.^30^ Colonoscopy with clinical pathology is regarded as the gold standard in CRC diagnosis, but they involve invasive and expensive procedures. In addition, the entire process of colonoscopy is a heavy burden on patients, and the observation results are sometimes biased owing to differences between operators, especially for early lesions, which reduces the accuracy of screening. Therefore, there is a great need for accurate and non‐invasive serum diagnostic tests for CRC and precancerous lesions.

The ITGB4‐encoded integrin β4 subunit, which is the laminin receptor, exclusively associates with the α6 subunit and may play a key role in the biology of infiltrating cancer.^31^, ^32^ Relevant studies have shown that ITGB4 plays a role in cell adhesion by binding to ECM adhesion proteins and transmitting signals that regulate cell function.^9^, ^33^, ^34^ ITGB4, which forms a dimer with integrin α6 (ITGA6), has been widely studied in carcinomas.^35^ During carcinoma progression, integrin α6β4 is released from hemidesmosomes, which allows it to be associated with the actin cytoskeleton.^36^ Here, it activates RhoA, leading to membrane ruffling, lamellae formation, and traction force generation and consequently promoting invasive and metastatic behavior.^37^

However, the molecular mechanism of ITGB4 in CRC is poorly understood. Our previous studies showed that ITGB4 is highly expressed in human CRC tissues and is associated with poor overall survival.^8^ Kajiji et al^38^ and Desgrosellier et al^10^ demonstrated that ITGB4 expression levels were significantly increased in a variety of malignancies. These findings together imply that ITGB4 plays a role in promoting CRC. To investigate the mechanism underlying the role of ITGB4 in CRC, the biological function of ITGB4 was examined in HCT116 (Figure S6) and SW480 (Figure S7) cell lines. The data indicated that the decreased expression of ITGB4 significantly inhibited the migration and invasion of CRC cells. Metastasis is a complex biological cascade that begins with the local invasion of tumor cells and continues with the migration of these cells to distant tissues that they eventually colonize.^39^ Thus, the results of this study demonstrate the value of ITGB4 being studied as a potential therapeutic target for CRC. However, the knockdown of ITGB4 had no significant effect on the proliferation and apoptosis of CRC cells in this study, which is different from the results of Hong et al,^40^ where ITGB4 promoted the proliferation of gastric cancer cells. Therefore, the phenotype of ITGB4 in different tumor cells appears to be more complicated than expected, and further research is needed.

We performed ITGB4 quantitative analysis on serum samples from 49 CRC patients and 367 HCs and found that ITGB4 could accurately discriminate CRC patients from normal individuals. In addition, this analysis showed that the sensitivity and specificity of ITGB4 for CRC diagnosis were superior to those of CEA, which is the only blood test for this disease worldwide.

This finding indicates that ITGB4 may have an extremely high potential for use in mass screening and diagnosis. Based on these data, we initially determined the threshold value of ITBG4 as a way of diagnosing CRC. Next, we tested the ITBG4 status of 1729 participants who underwent CRC screening in a high‐risk population in China. The sensitivity and specificity of the ITGB4 method were 79.59% and 60.65%, respectively, with an AUC of 0.7216. The results show that the use of ITGB4 as a CRC biomarker is not only highly sensitive, but also has a great practical value. In addition, further elucidation of underlying mechanisms may provide potential targets for therapeutic interventions for CRC as well as methods to prevent CRC.

Our current study found that ITGB4 has a relatively high and stable diagnostic value for CRC in the Chinese population. However, there are some limitations that need to be addressed. Biopsy samples were used for pathological diagnosis, and some information (such as depth of infiltration and lymph node metastasis) was not available. Second, the clinical cases come from a single cohort; thus, in order to improve the accuracy of diagnosis, a multicenter study is needed to optimize the cutoff value. Third, this was not a randomized controlled study; inevitably, there was selection bias. In this prospective nested case–control study, there were more patients with stage I and II CRC (I & II: III & IV = 2.08:1), and we found that the serum ITGB4 levels of stage III and IV CRC patients were higher than those of stage I and II CRC patients. Therefore, the diagnostic efficacy of ITGB4 will improve after correcting for selection bias. Finally, this study did not discuss the diagnostic efficacy of ITGB4 combined with FIT, and further studies are needed to analyze the diagnostic efficacy of ITGB4 combined with FIT in CRC.

In summary, our findings prove the accurate and noninvasive diagnostic value of ITGB4 for CRC, and ITGB4 can be applied in the detection of asymptomatic cases of CRC. The results of this study support the establishment of large‐scale randomized clinical trials to verify the clinical applicability of ITGB4 in the diagnosis of CRC.

## CONFLICT OF INTEREST

The authors declare no potential conflict of interest.

## AUTHOR CONTRIBUTION

Data curation: JW, MW, MX, and SH; Formal analysis: MX, CL, and ML; Funding acquisition: WY and ZZ; Methodology: XJ, JW and ZZ; Project administration: XJ, WY and ZZ; Resources & Software: XS and WY; Write and review manuscript: XJ and MW.

## Supporting information

Fig S1

Fig S2

Fig S3

Fig S4

Fig S5

Fig S6

Fig S7

Fig S8

Table S1

Table S2

Table S3

Table S4

Table S5

Supplementary Material

## Data Availability

Data sharing is not applicable.
