# Supplementary figures and images for: ITGB4 as a novel serum diagnosis biomarker and potential therapeutic target for colorectal cancer
Source: Cancer Med. 2021 Aug 20;10(19):6823–34. doi: 10.1002/cam4.4216 (PMC8495272; doi:10.1002/cam4.4216)

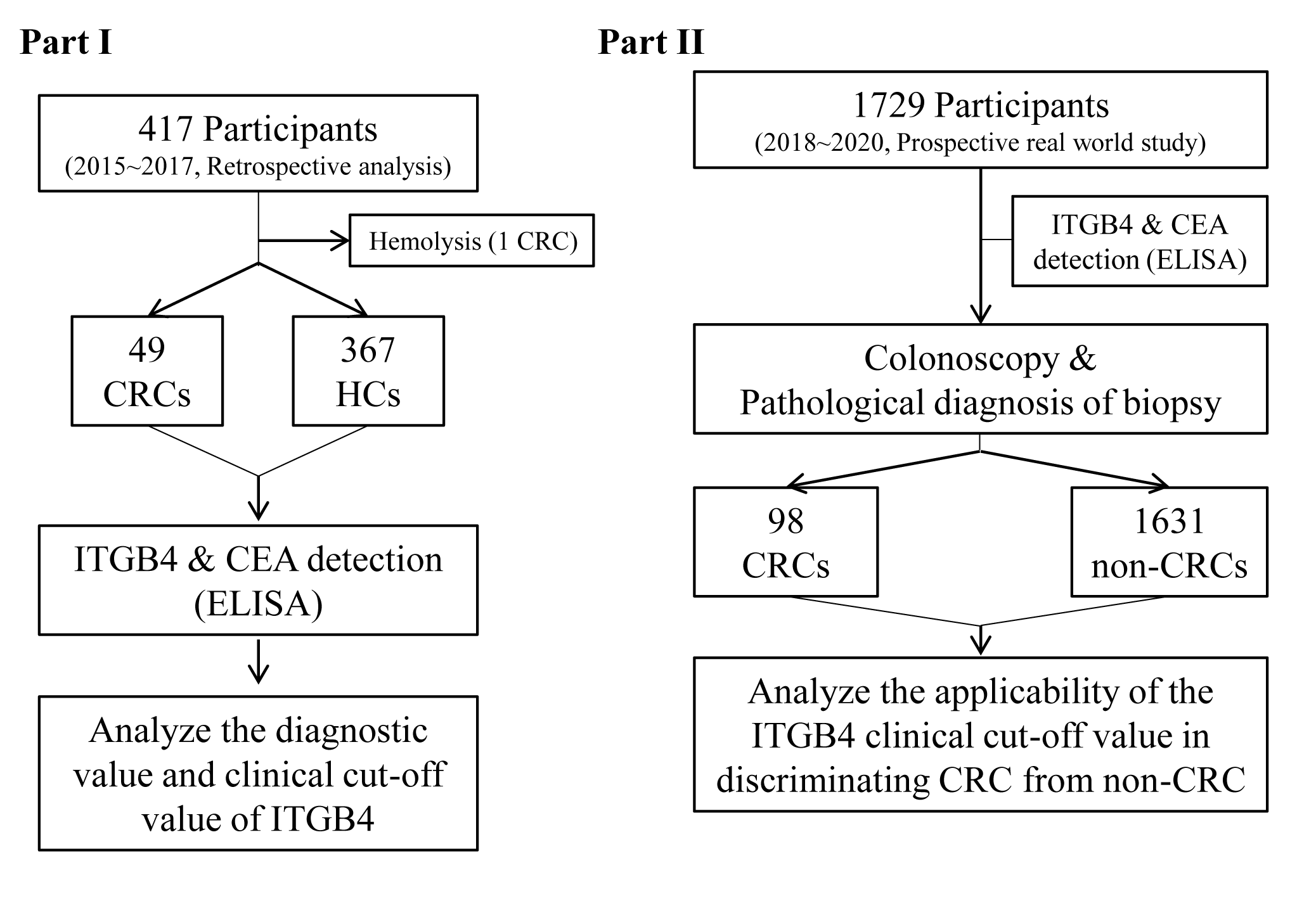

Supplement: Supplementary file 1 — Fig S1 [file CAM4-10-6823-s007.tif]

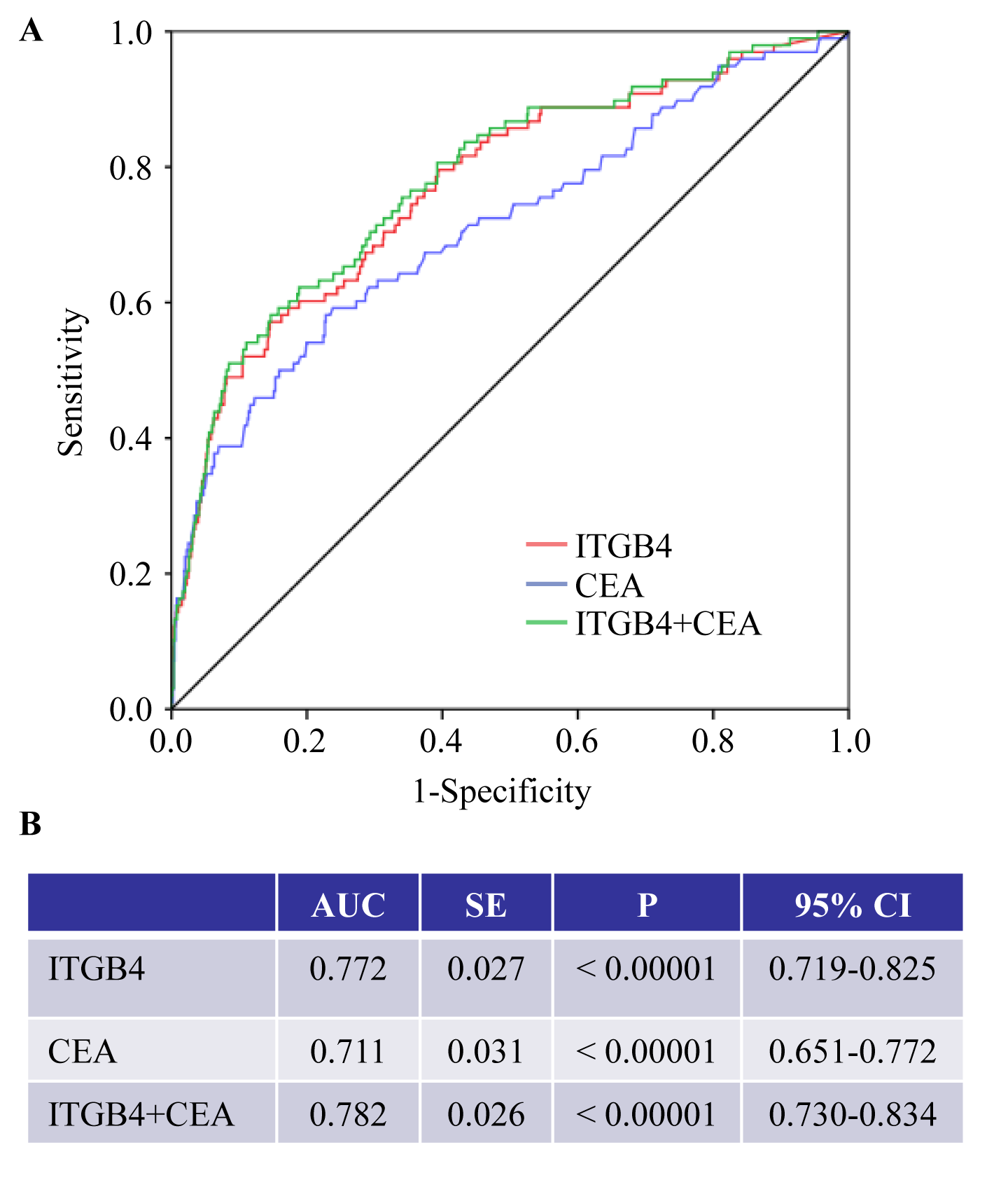

Supplement: Supplementary file 2 — Fig S2 [file CAM4-10-6823-s004.tiff]

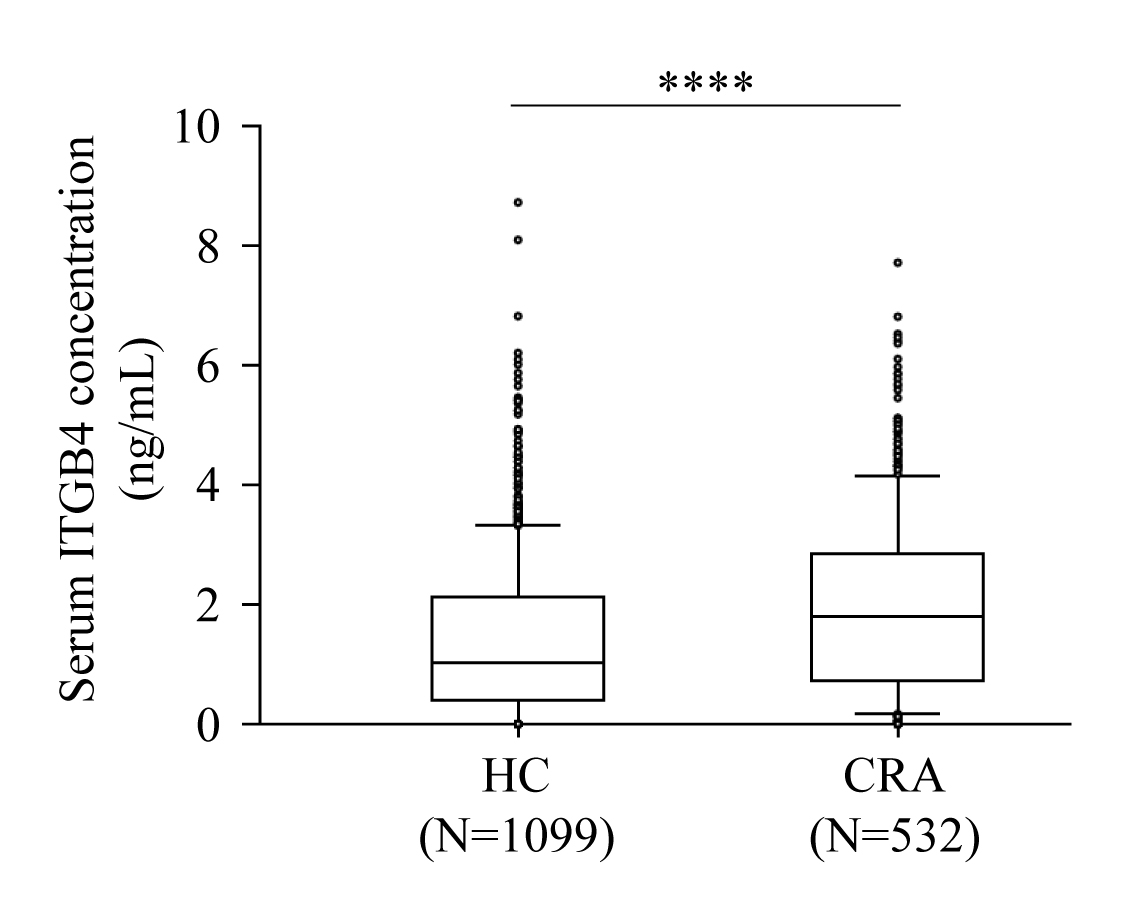

Supplement: Supplementary file 3 — Fig S3 [file CAM4-10-6823-s009.tiff]

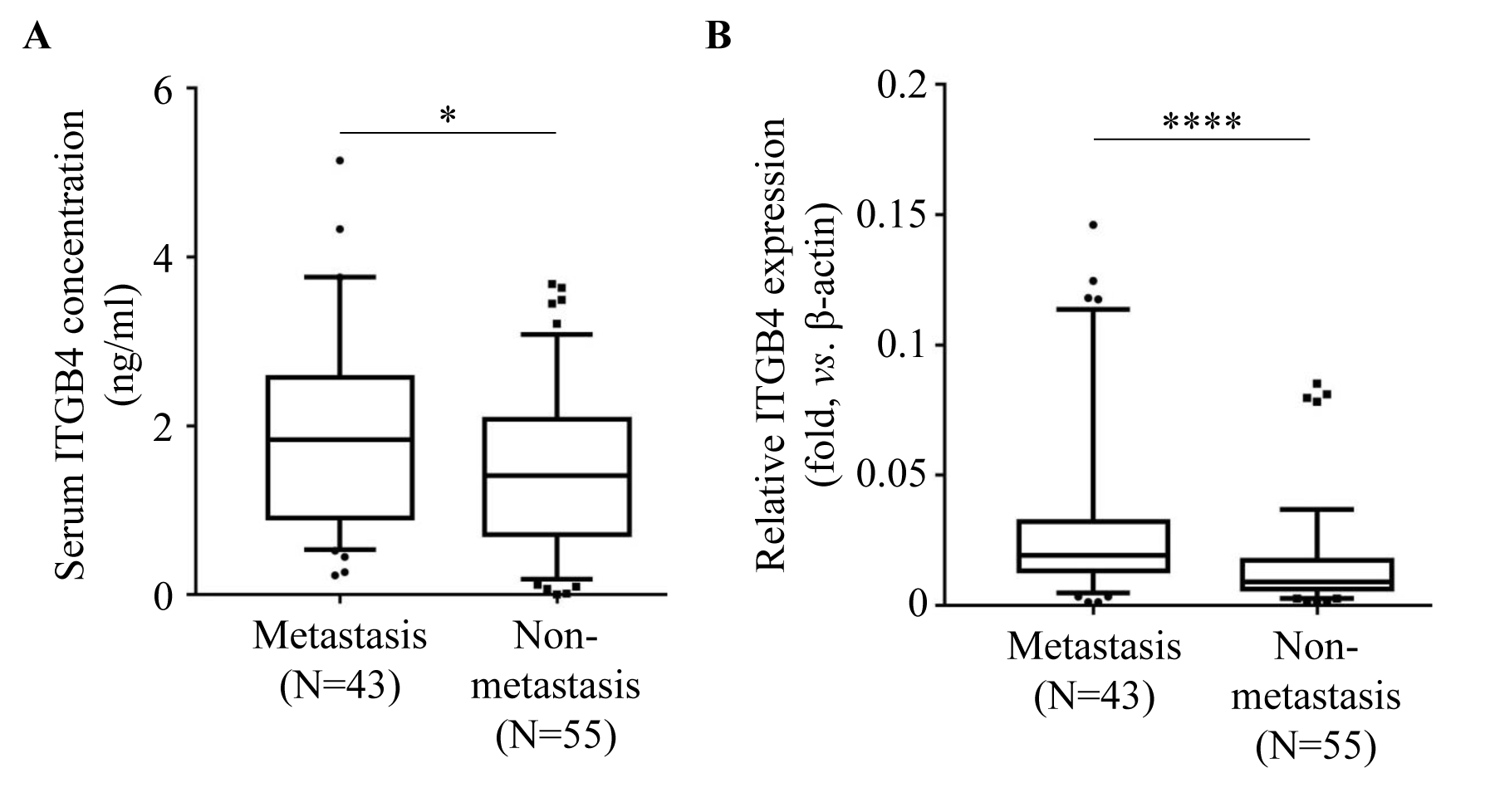

Supplement: Supplementary file 4 — Fig S4 [file CAM4-10-6823-s001.tif]

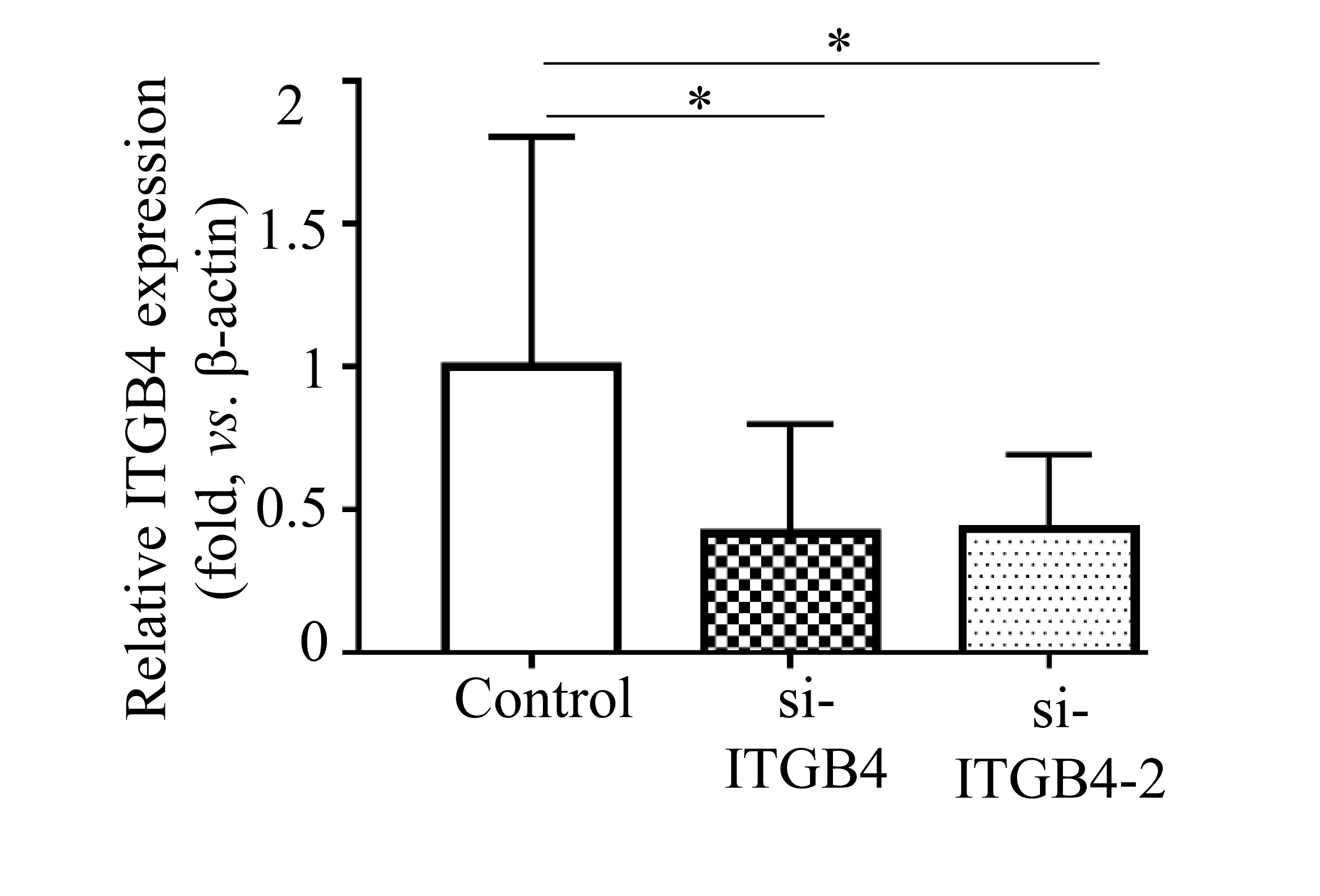

Supplement: Supplementary file 5 — Fig S5 [file CAM4-10-6823-s003.png]

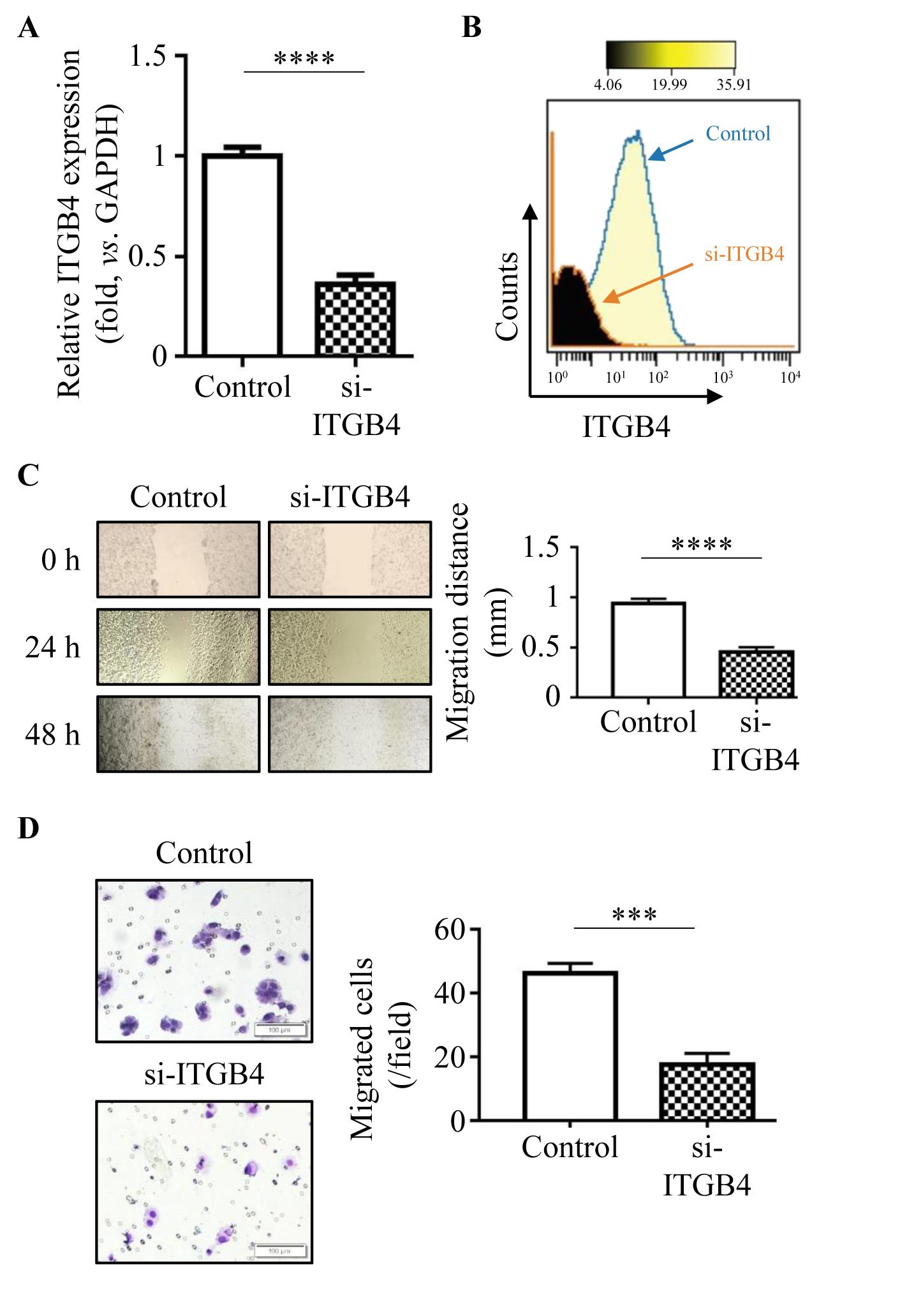

Supplement: Supplementary file 6 — Fig S6 [file CAM4-10-6823-s013.tiff]

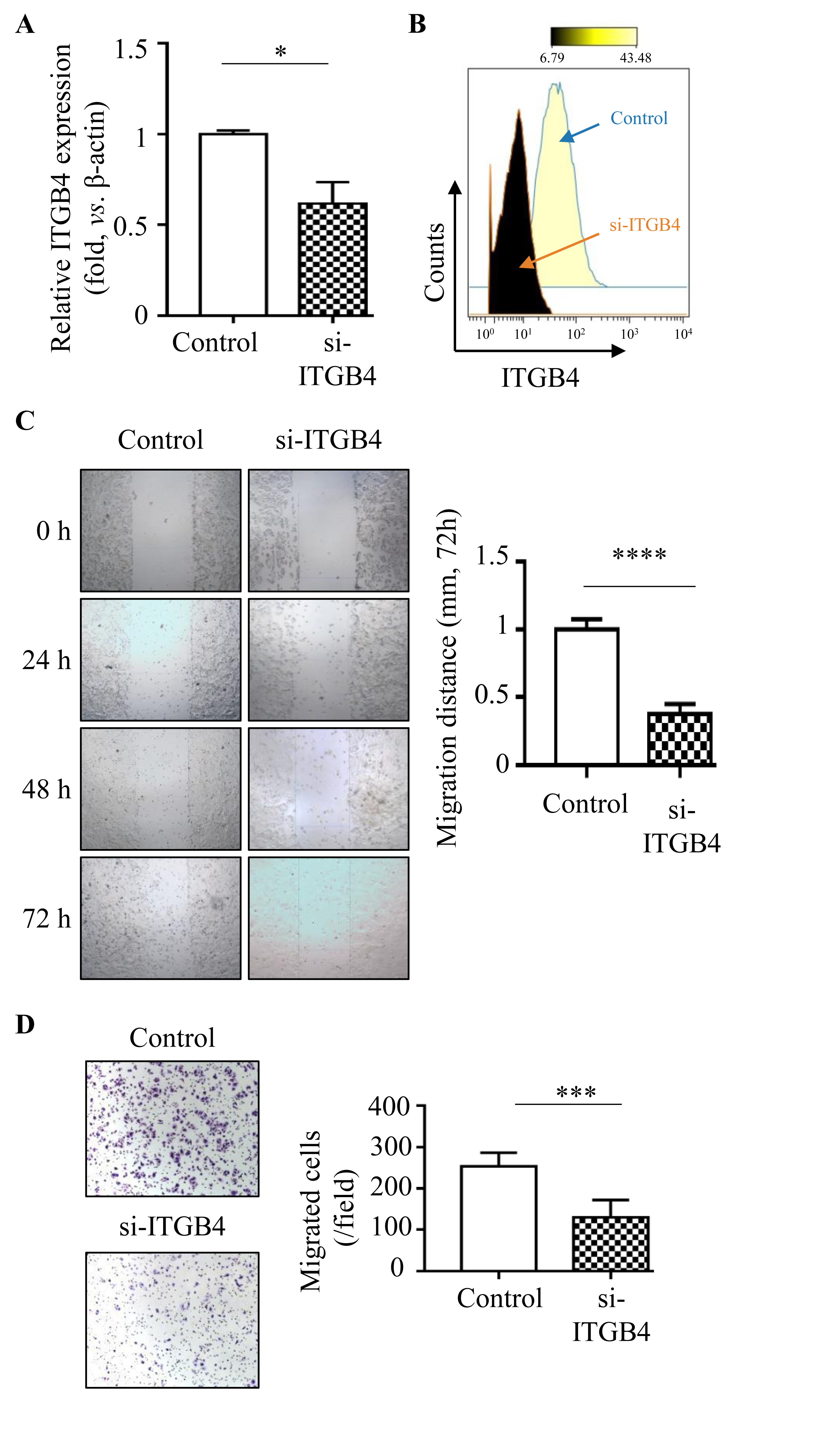

Supplement: Supplementary file 7 — Fig S7 [file CAM4-10-6823-s011.tif]

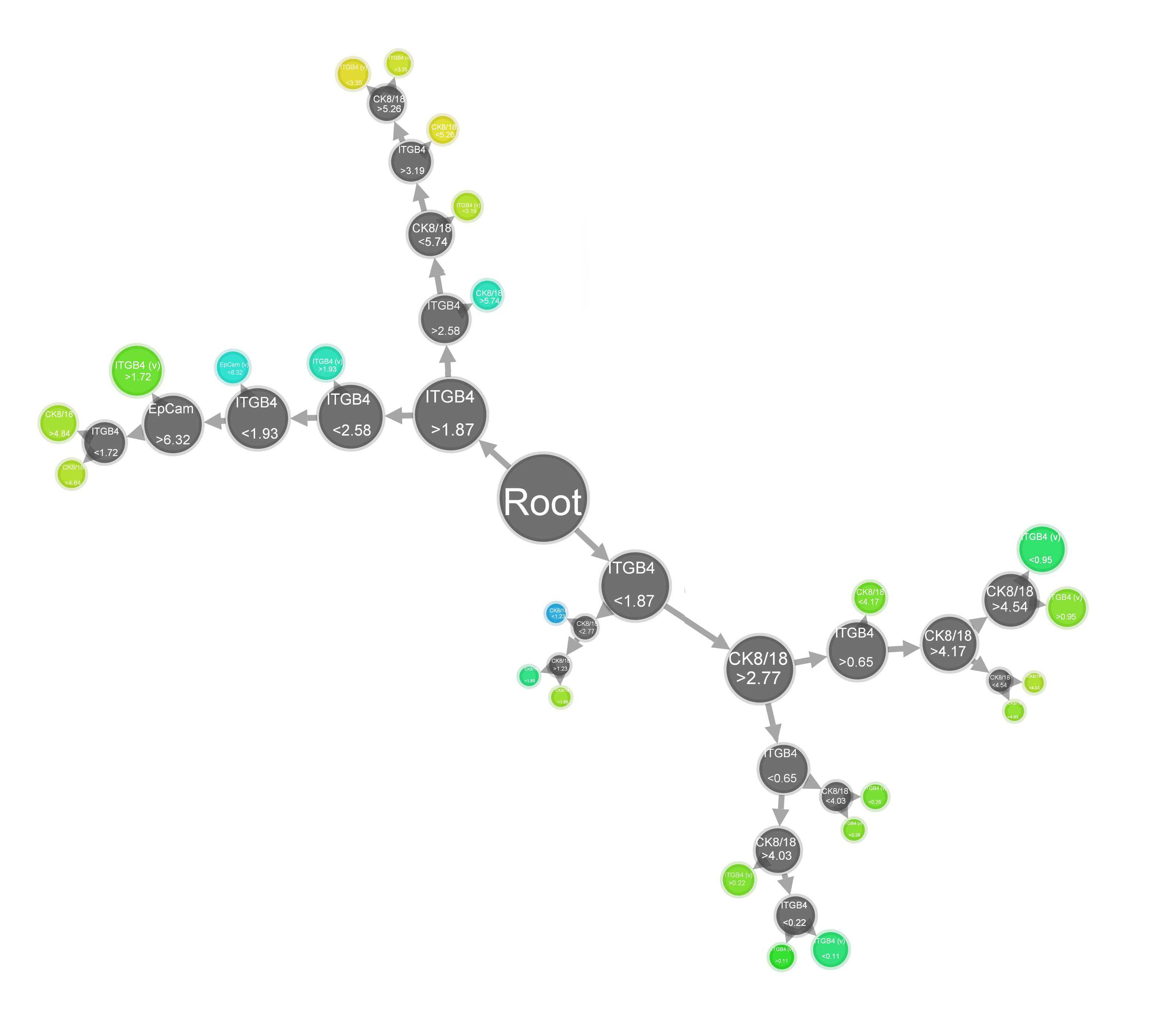

Supplement: Supplementary file 8 — Fig S8 [file CAM4-10-6823-s002.tif]
