# Supplementary material for: ITGB4 as a novel serum diagnosis biomarker and potential therapeutic target for colorectal cancer
Source: Cancer Med. 2021 Aug 20;10(19):6823–34. doi: 10.1002/cam4.4216 (PMC8495272; doi:10.1002/cam4.4216)
Supplement: Supplementary file 9 — Table S1 [file CAM4-10-6823-s005.docx]

Supplemental Table 1. Clinical characteristics of patients in Part I

| **Diagnosis^a^** | **Variable** | **Patients** | **ITGB4 Concentration** | **ITGB4 diagnostic efficiency^b^** | | **P values^c^** | **P values^d^** |
| --- | --- | --- | --- | --- | --- | --- | --- |
|  |  |  |  | Number of positive results | Positive rate % |  |  |
| CRC | Age | 68.00 (56.00-75.00) | |  |  |  | < 0.00001 |
|  | Gender | 49 |  |  |  | 0.856 | 0.04 |
|  | Male | 28 | 1.24 (0.79-2.09) | 23 | 82.14 |  |  |
|  | Female | 21 | 1.62 (0.68-2.24) | 16 | 76.19 |  |  |
|  | Histological grade |  |  |  |  | 0.047 |  |
|  | Poorly | 3 | 2.24 (1.94) | 3 | 100 |  |  |
|  | Moderately | 18 | 1.24 (0.68-2.16) | 14 | 77.78 |  |  |
|  | Well | 17 | 0.99 (0.63-1.78) | 12 | 70.59 |  |  |
|  | T-stage |  |  |  |  | 0.715 |  |
|  | ≤ T2 | 18 | 1.05 (0.69-1.90) | 14 | 77.78 |  |  |
|  | ≥ T3 | 19 | 1.26 (0.60-2.07) | 14 | 73.68 |  |  |
|  | N-stage |  |  |  |  | 0.856 |  |
|  | N0 | 33 | 1.21 (0.68-2.02) | 25 | 75.76 |  |  |
|  | ≥ N1 | 5 | 1.26 (0.37-2.56) | 4 | 80.00 |  |  |
|  | M-stage |  |  |  |  | 0.753 |  |
|  | M0 | 44 | 1.24 (0.73-2.07) | 35 | 79.55 |  |  |
|  | M1 | 3 | 1.94 (0.27) | 2 | 66.67 |  |  |
|  | TNM stage |  |  |  |  | 1.000 |  |
|  | I+II | 30 | 1.16 (0.69-2.00) | 23 | 76.67 |  |  |
|  | III+IV | 7 | 1.26 (0.27-2.35) | 5 | 71.43 |  |  |
| HC | Age | 55.00 (48.00-63.00) | |  |  |  |  |
|  | Gender | 367 |  |  |  | 0.019 |  |
|  | Male | 153 | 0.61 (0.25-1.10) | 71 | 46.41 |  |  |
|  | Female | 214 | 0.43 (0.14-0.84) | 68 | 31.78 |  |  |

^a^ Pathological diagnosis of endoscopic biopsy specimens

^b^ Diagnostic efficiency ITGB4 of the ITGB4 clinical cut-off value (0.70 ng/mL)

^c^ Statistical analysis of ITGB4 concentration within the same group

^d^ Statistical analysis between CRC group and HC group
