# Supplementary material for: ITGB4 as a novel serum diagnosis biomarker and potential therapeutic target for colorectal cancer
Source: Cancer Med. 2021 Aug 20;10(19):6823–34. doi: 10.1002/cam4.4216 (PMC8495272; doi:10.1002/cam4.4216)
Supplement: Supplementary file 10 — Table S2 [file CAM4-10-6823-s012.docx]

Supplemental Table 2. Clinical characteristics of patients in Part II

| **Diagnosis^a^** | | **Variable** | **Patients** | **ITGB4 Concentration** | | **ITGB4 diagnostic efficiency^b^** | | | **P values^c^** | **P values^d^** |
| --- | --- | --- | --- | --- | --- | --- | --- | --- | --- | --- |
|  |  |  |  |  |  | Number of positive results | | Positive rate % |  |  |
| CRC | | Age | 66.00 (55.75-73.00) | | |  | |  |  | < 0.00001 |
|  | | Gender | 98 |  | |  | |  | 0.406 | < 0.00001 |
|  | | Male | 57 | 1.41 (0.78-2.10) | | 47 | | 82.46 |  |  |
|  | | Female | 41 | 1.80 (0.73-2.36) | | 32 | | 78.05 |  |  |
|  | | Histological grade |  |  | |  | |  | 0.465 |  |
|  | | Poorly | 13 | 1.94 (0.91-2.35) | | 10 | | 76.92 |  |  |
|  | | Moderately | 29 | 1.08 (0.57-2.19) | | 21 | | 72.41 |  |  |
|  | | Well | 35 | 1.62 (0.72-1.97) | | 28 | | 80.00 |  |  |
|  | | T-stage |  |  | |  | |  | 0.625 |  |
|  | | ≤ T2 | 36 | 1.67 (0.82-2.09) | | 30 | | 83.33 |  |  |
|  | | ≥ T3 | 40 | 1.35 (0.59-2.11) | | 28 | | 70.00 |  |  |
|  | | N-stage |  |  | |  | |  | 0.864 |  |
|  | | N0 | 56 | 1.41 (0.73-2.08) | | 44 | | 78.57 |  |  |
|  | | ≥ N1 | 21 | 1.63 (0.55-2.42) | | 15 | | 71.43 |  |  |
|  | | M-stage |  |  | |  | |  | 0.399 |  |
|  | | M0 | 83 | 1.62 (0.78-2.23) | | 68 | | 81.93 |  |  |
|  | | M1 | 10 | 1.03 (0.50-2.15) | | 6 | | 60.00 |  |  |
|  | | TNM stage |  |  | |  | |  | 0.802 |  |
|  | | I+II | 52 | 1.41 (0.73-2.08) | | 41 | | 78.85 |  |  |
|  | | III+IV | 25 | 1.63 (0.55-2.42) | | 18 | | 72.00 |  |  |
| Non-  CRC | CRA | Age | 59.00 (50.00-65.00) | | | | | | | |
|  |  | Gender | 532 |  |  | |  | | 0.619 |  |
|  |  | Male | 338 | 0.84 (0.35-1.40) | 196 | | 57.99 | |  |  |
|  |  | Female | 194 | 0.91 (0.39-1.44) | 113 | | 58.25 | |  |  |
|  | HC | Age | 55.00 (48.00-63.00) | | | | | | |  |
|  |  | Gender | 1099 |  |  | |  | | 0.001 |  |
|  |  | Male | 465 | 0.47 (0.20-1.06) | 185 | | 39.78 | |  |  |
|  |  | Female | 634 | 0.39 (0.15-0.77) | 177 | | 27.92 | |  |  |

^a^ Pathological diagnosis of endoscopic biopsy specimens

^b^ Diagnostic efficiency ITGB4 of the ITGB4 clinical cut-off value (0.70 ng/mL)

^c^ Statistical analysis of ITGB4 concentration within the same group

^d^ Statistical analysis between CRC group and HC group
