# Supplementary material for: ITGB4 as a novel serum diagnosis biomarker and potential therapeutic target for colorectal cancer
Source: Cancer Med. 2021 Aug 20;10(19):6823–34. doi: 10.1002/cam4.4216 (PMC8495272; doi:10.1002/cam4.4216)
Supplement: Supplementary file 11 — Table S3 [file CAM4-10-6823-s010.docx]

Supplementary Table 3. The information of metal conjugated antibodies panel

| ***Metal/channels*** | ***Parameters/antibodies*** |
| --- | --- |
| Pd102Di | Barcode |
| Pd104Di | Barcode |
| Pd105Di | Barcode |
| Pd106Di | Barcode |
| Pd108Di | Barcode |
| Pd110Di | Barcode |
| Ce140Di | Beads |
| Pr141Di | EpCam |
| Ce142Di | Beads |
| Nd144Di | IL-4 |
| Nd148Di | GAL |
| Nd150Di | MIP-1 |
| Eu151Di | IL-5 |
| Sm152Di | TNFα |
| Eu153Di | Beads |
| Sm154Di | CD45 |
| Gd156Di | IL-6 |
| Gd158Di | IL-2 |
| Gd160Di | CD14 |
| Dy164Di | IL-17 |
| Ho165Di | Beads |
| Er168Di | IFNg |
| Er170Di | CD3 |
| Yb171Di | GranzymB |
| Yb173Di | ITGB4 |
| Yb174Di | CK8/18 |
| Lu175Di | Perforin |
| Ir191Di | DNA |
| Ir193Di | DNA |
| Pt195Di | Live/Dead |
