# Supplementary material for: ITGB4 as a novel serum diagnosis biomarker and potential therapeutic target for colorectal cancer
Source: Cancer Med. 2021 Aug 20;10(19):6823–34. doi: 10.1002/cam4.4216 (PMC8495272; doi:10.1002/cam4.4216)
Supplement: Supplementary file 12 — Table S4 [file CAM4-10-6823-s008.docx]

Supplementary Table 4. The information of 34 potential CRC diagnosis associated proteins

| **Gene Symbol** | **Gene Name** | **Fold change^a^** |
| --- | --- | --- |
| KRT5 | keratin 5 | 1046.7 |
| CST1 | cystatin SN | 496.0 |
| TGM2 | transglutaminase 2 | 296.9 |
| SLC7A5 | solute carrier family 7 member 5 | 162.6 |
| FGF19 | fibroblast growth factor 19 | 151.8 |
| SLC2A3 | solute carrier family 2 member 3 | 122.4 |
| PRF1 | perforin 1 | 116.3 |
| DHRS2 | dehydrogenase/reductase 2 | 103.4 |
| ANO1 | anoctamin 1 | 67.6 |
| CST4 | cystatin S | 52.7 |
| KRT13 | keratin 13 | 49.7 |
| ALDH1A3 | aldehyde dehydrogenase 1 family member A3 | 49.4 |
| PPL | periplakin | 42.0 |
| ITGB4 | integrin subunit beta 4 | 35.2 |
| PSAT1 | phosphoserine aminotransferase 1 | 34.5 |
| SLC1A5 | solute carrier family 1 member 5 | 33.8 |
| INHBB | inhibin beta B subunit | 28.7 |
| LAMA5 | laminin subunit alpha 5 | 24.8 |
| MIA | melanoma inhibitory activity | 22.4 |
| LIF | leukemia inhibitory factor | 20.6 |
| EPS8L2 | EPS8 like 2 | 15.8 |
| TNFRSF6B | TNF receptor superfamily member 6b | 15.5 |
| FKBP4 | FK506 binding protein 4 | 11.8 |
| ITGB5 | integrin subunit beta 5 | 11.5 |
| WNT5A | Wnt family member 5A | 11.0 |
| MDK | midkine | 10.6 |
| ACTN4 | actinin alpha 4 | 9.3 |
| PLEC | plectin | 8.3 |
| CTDSPL | CTD small phosphatase like | 7.4 |
| CSF2 | colony stimulating factor 2 | 7.0 |
| PLOD3 | procollagen-lysine,2-oxoglutarate 5-dioxygenase 3 | 6.7 |
| PLP2 | proteolipid protein 2 | 5.7 |
| COPS6 | COP9 signalosome subunit 6 | 5.2 |
| KRT18 | keratin 18 | 5.2 |

^a^ Fold change was calculated by the results of BIOGPS (http://ds.biogps.org/?dataset=GSE1133&gene=3691, Dataset: GeneAtlas U133A, gcrma)
