# Supplementary material for: ITGB4 as a novel serum diagnosis biomarker and potential therapeutic target for colorectal cancer
Source: Cancer Med. 2021 Aug 20;10(19):6823–34. doi: 10.1002/cam4.4216 (PMC8495272; doi:10.1002/cam4.4216)
Supplement: Supplementary file 13 — Table S5 [file CAM4-10-6823-s014.docx]

Supplementary Table 5. Performance of ITGB4 or CEA alone and in combination with eachother for colorectal cancer diagnosis in distinguishing CRC (N=98) from HC (N=1099)

|  | **ITGB4 (cut-off=0.7 ng/mL)** | | **CEA**  **(5 ng/mL)** | **ITGB4 (cut-off=1.6 ng/mL)** | |
| --- | --- | --- | --- | --- | --- |
|  | **ITGB4** | **ITGB4+CEA** |  | **ITGB4** | **ITGB4+CEA** |
| **Ture Positive (N)** | **78** | **87** | **32** | **51** | **70** |
| **False Positive (N)** | **439** | **465** | **47** | **116** | **160** |
| **False Negative (N)** | **20** | **11** | **66** | **47** | **28** |
| **Ture Negative (N)** | **660** | **634** | **1052** | **983** | **939** |
| **Sensitivity** | **79.6%** | **88.8%** | **32.7%** | **52.0%** | **71.4%** |
| **Specificity** | **60.1%** | **57.7%** | **95.7%** | **89.4%** | **85.4%** |
