# Supplementary material for: ITGB4 as a novel serum diagnosis biomarker and potential therapeutic target for colorectal cancer
Source: Cancer Med. 2021 Aug 20;10(19):6823–34. doi: 10.1002/cam4.4216 (PMC8495272; doi:10.1002/cam4.4216)
Supplement: Supplementary file 14 — Supplementary Material [file CAM4-10-6823-s006.docx]

Supplementary Figure 1


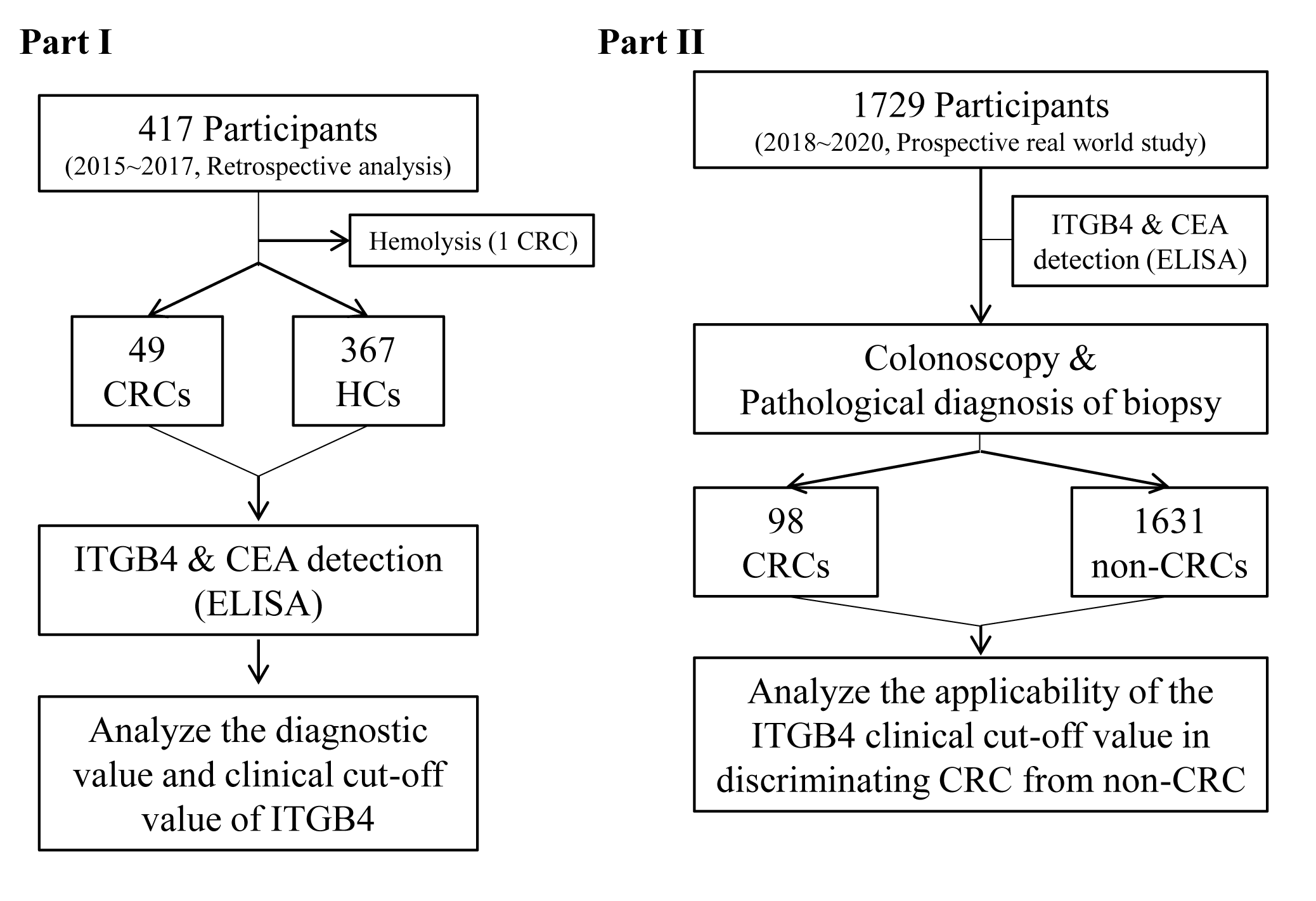


Supplementary Figure 2


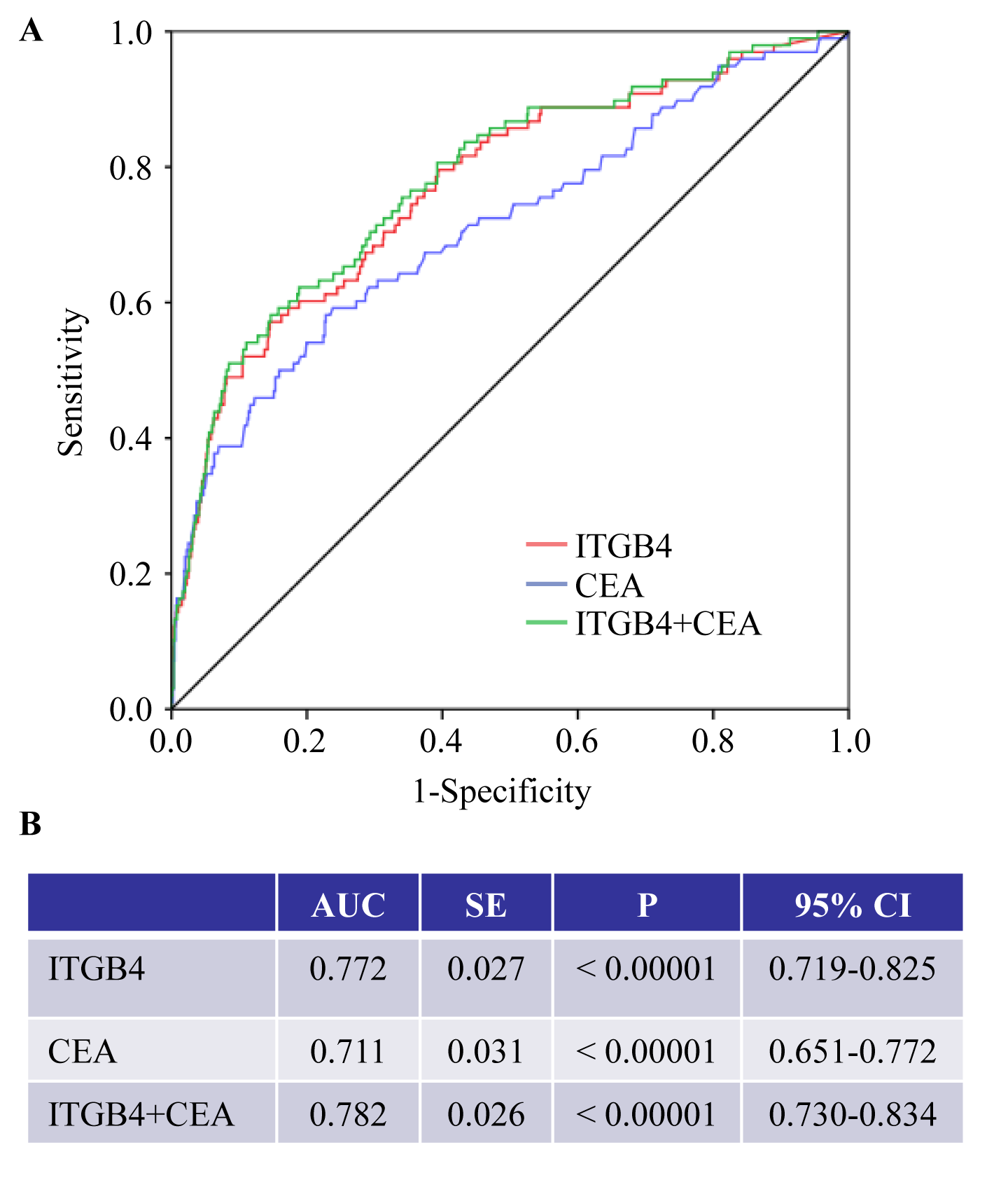


Supplementary Figure 3


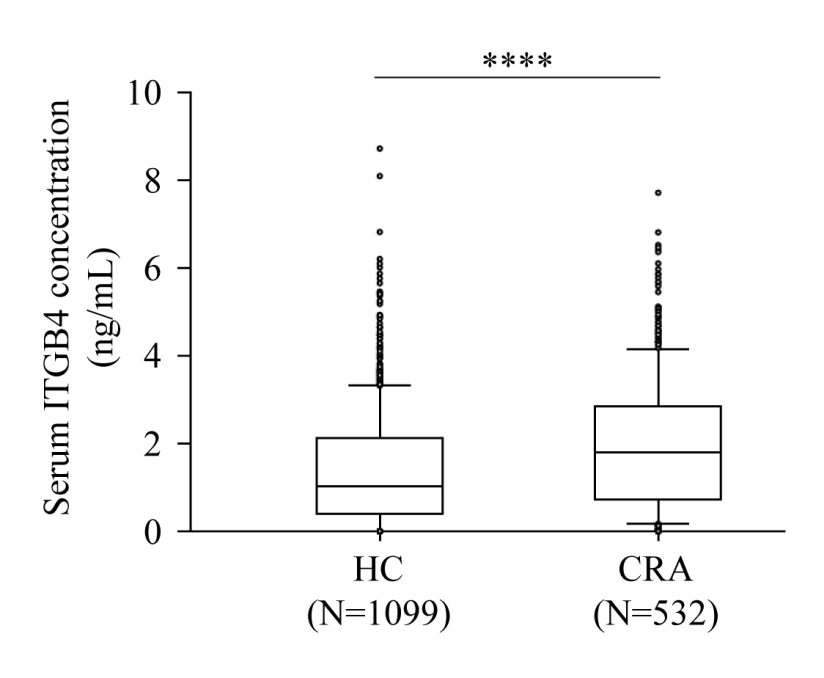


Supplementary Figure 4


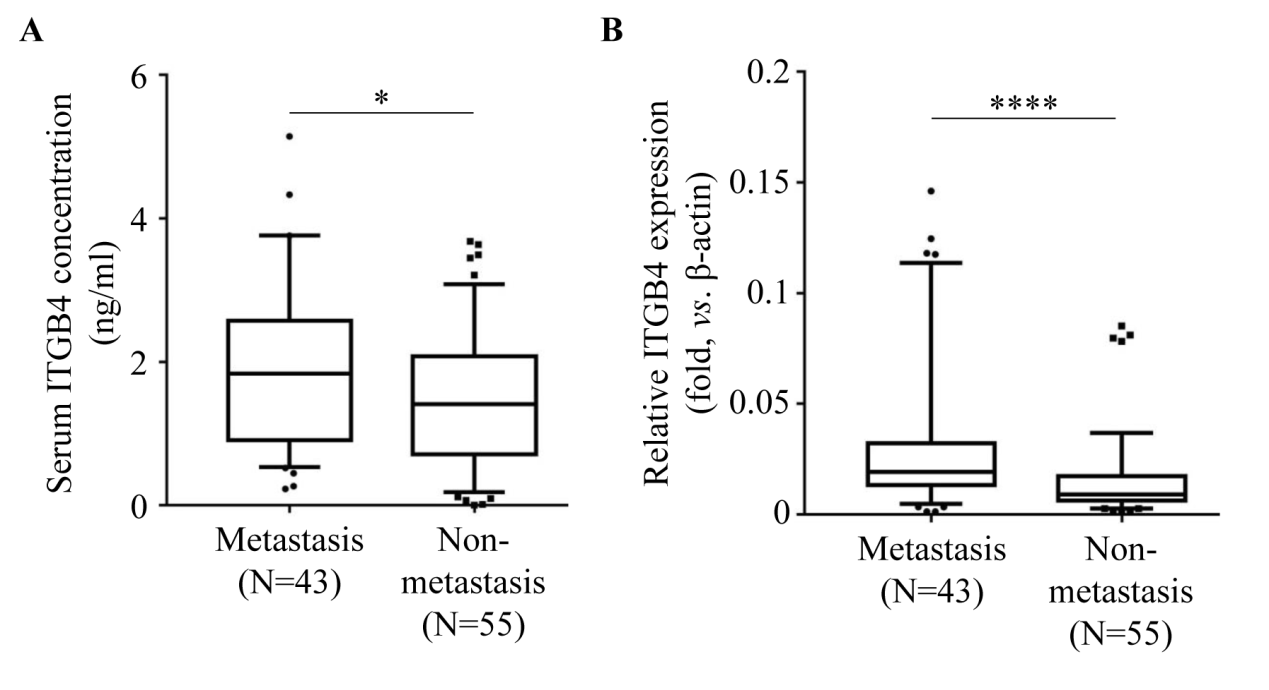


Supplementary Figure 5


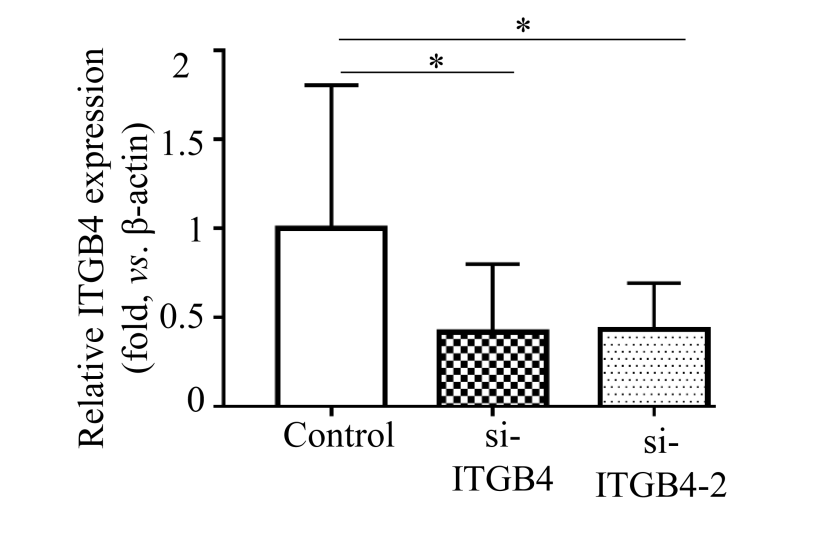


Supplementary Figure 6


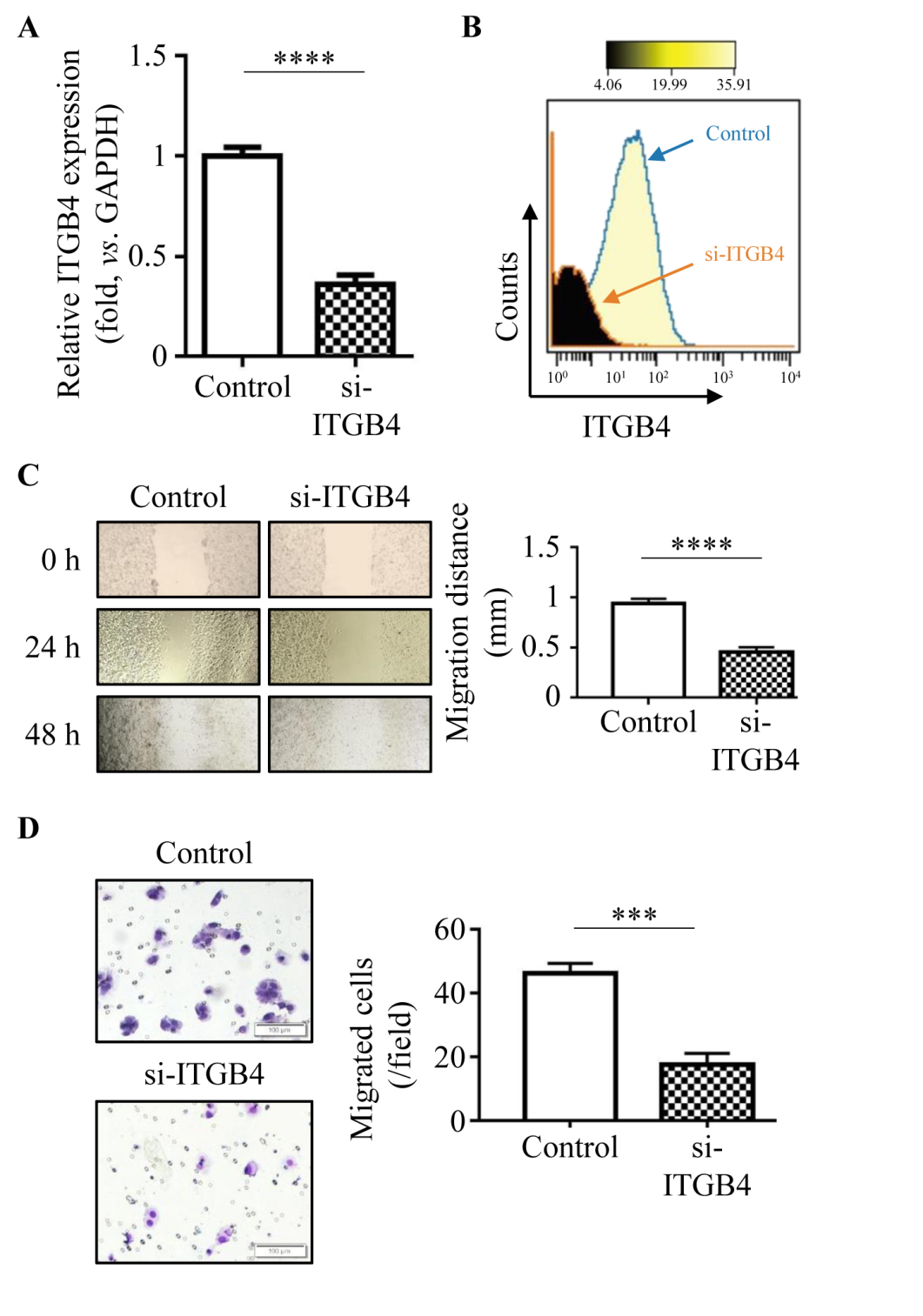


Supplementary Figure 7


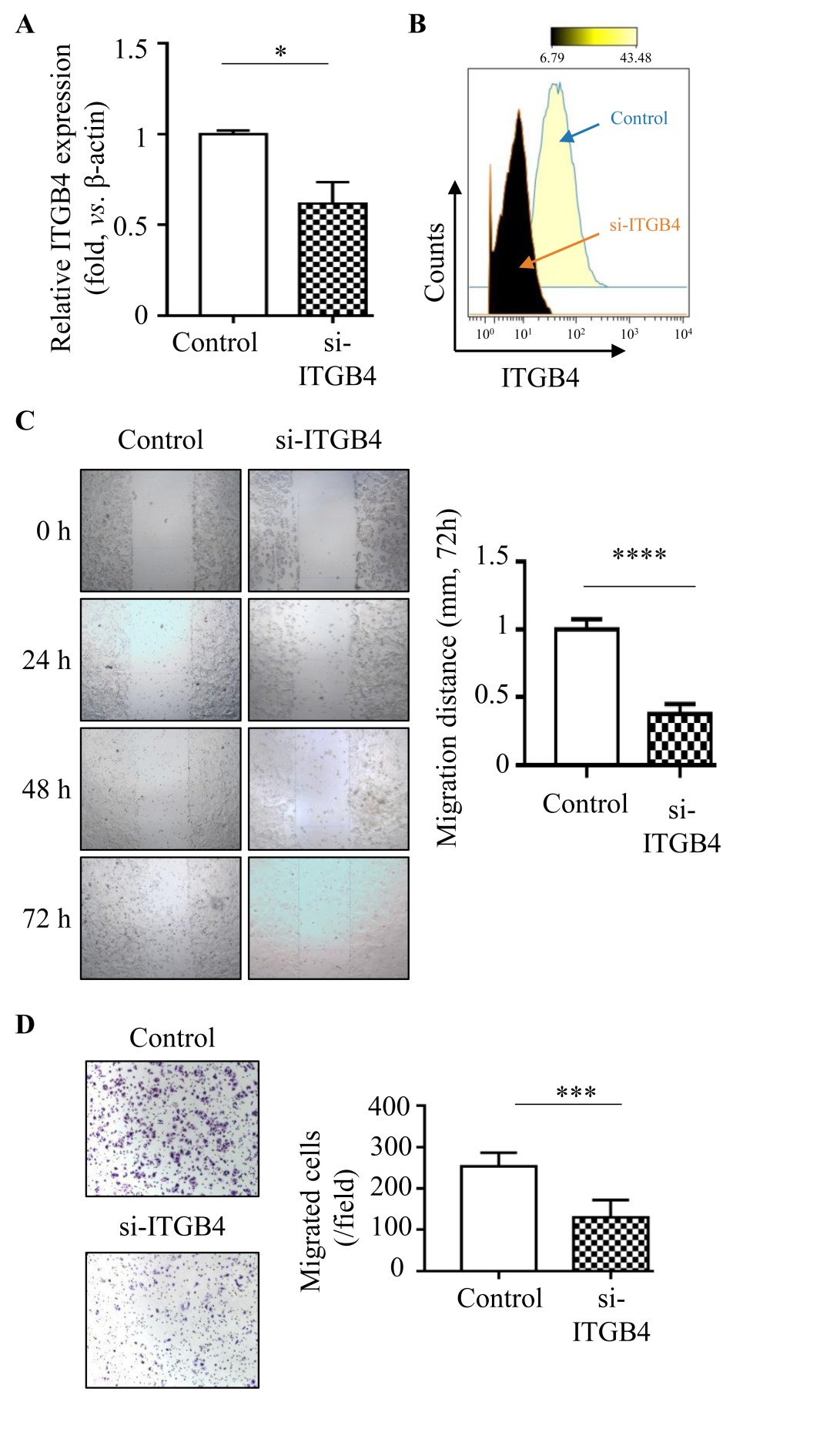


Supplementary Figure 8


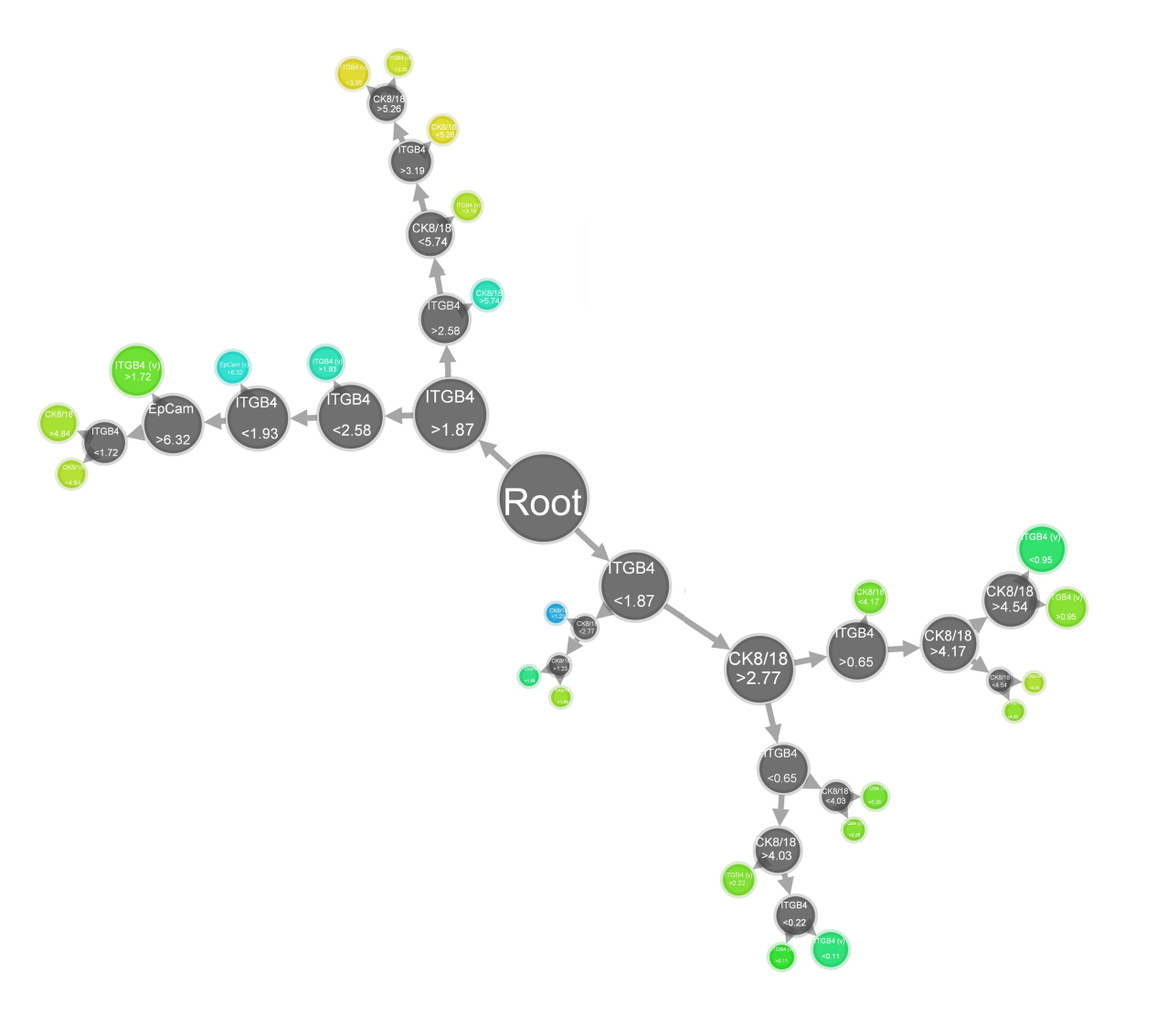


Supplemental Table 1. Clinical characteristics of patients in Part I

| **Diagnosis^a^** | **Variable** | **Patients** | **ITGB4 Concentration** | **ITGB4 diagnostic efficiency^b^** | | **P values^c^** | **P values^d^** |
| --- | --- | --- | --- | --- | --- | --- | --- |
|  |  |  |  | Number of positive results | Positive rate % |  |  |
| CRC | Age | 68.00 (56.00-75.00) | |  |  |  | < 0.00001 |
|  | Gender | 49 |  |  |  | 0.856 | 0.04 |
|  | Male | 28 | 1.24 (0.79-2.09) | 23 | 82.14 |  |  |
|  | Female | 21 | 1.62 (0.68-2.24) | 16 | 76.19 |  |  |
|  | Histological grade |  |  |  |  | 0.047 |  |
|  | Poorly | 3 | 2.24 (1.94) | 3 | 100 |  |  |
|  | Moderately | 18 | 1.24 (0.68-2.16) | 14 | 77.78 |  |  |
|  | Well | 17 | 0.99 (0.63-1.78) | 12 | 70.59 |  |  |
|  | T-stage |  |  |  |  | 0.715 |  |
|  | ≤ T2 | 18 | 1.05 (0.69-1.90) | 14 | 77.78 |  |  |
|  | ≥ T3 | 19 | 1.26 (0.60-2.07) | 14 | 73.68 |  |  |
|  | N-stage |  |  |  |  | 0.856 |  |
|  | N0 | 33 | 1.21 (0.68-2.02) | 25 | 75.76 |  |  |
|  | ≥ N1 | 5 | 1.26 (0.37-2.56) | 4 | 80.00 |  |  |
|  | M-stage |  |  |  |  | 0.753 |  |
|  | M0 | 44 | 1.24 (0.73-2.07) | 35 | 79.55 |  |  |
|  | M1 | 3 | 1.94 (0.27) | 2 | 66.67 |  |  |
|  | TNM stage |  |  |  |  | 1.000 |  |
|  | I+II | 30 | 1.16 (0.69-2.00) | 23 | 76.67 |  |  |
|  | III+IV | 7 | 1.26 (0.27-2.35) | 5 | 71.43 |  |  |
| HC | Age | 55.00 (48.00-63.00) | |  |  |  |  |
|  | Gender | 367 |  |  |  | 0.019 |  |
|  | Male | 153 | 0.61 (0.25-1.10) | 71 | 46.41 |  |  |
|  | Female | 214 | 0.43 (0.14-0.84) | 68 | 31.78 |  |  |

^a^ Pathological diagnosis of endoscopic biopsy specimens

^b^ Diagnostic efficiency ITGB4 of the ITGB4 clinical cut-off value (0.70 ng/mL)

^c^ Statistical analysis of ITGB4 concentration within the same group

^d^ Statistical analysis between CRC group and HC group

Supplemental Table 2. Clinical characteristics of patients in Part II

| **Diagnosis^a^** | | **Variable** | **Patients** | **ITGB4 Concentration** | | **ITGB4 diagnostic efficiency^b^** | | | **P values^c^** | **P values^d^** |
| --- | --- | --- | --- | --- | --- | --- | --- | --- | --- | --- |
|  |  |  |  |  |  | Number of positive results | | Positive rate % |  |  |
| CRC | | Age | 66.00 (55.75-73.00) | | |  | |  |  | < 0.00001 |
|  | | Gender | 98 |  | |  | |  | 0.406 | < 0.00001 |
|  | | Male | 57 | 1.41 (0.78-2.10) | | 47 | | 82.46 |  |  |
|  | | Female | 41 | 1.80 (0.73-2.36) | | 32 | | 78.05 |  |  |
|  | | Histological grade |  |  | |  | |  | 0.465 |  |
|  | | Poorly | 13 | 1.94 (0.91-2.35) | | 10 | | 76.92 |  |  |
|  | | Moderately | 29 | 1.08 (0.57-2.19) | | 21 | | 72.41 |  |  |
|  | | Well | 35 | 1.62 (0.72-1.97) | | 28 | | 80.00 |  |  |
|  | | T-stage |  |  | |  | |  | 0.625 |  |
|  | | ≤ T2 | 36 | 1.67 (0.82-2.09) | | 30 | | 83.33 |  |  |
|  | | ≥ T3 | 40 | 1.35 (0.59-2.11) | | 28 | | 70.00 |  |  |
|  | | N-stage |  |  | |  | |  | 0.864 |  |
|  | | N0 | 56 | 1.41 (0.73-2.08) | | 44 | | 78.57 |  |  |
|  | | ≥ N1 | 21 | 1.63 (0.55-2.42) | | 15 | | 71.43 |  |  |
|  | | M-stage |  |  | |  | |  | 0.399 |  |
|  | | M0 | 83 | 1.62 (0.78-2.23) | | 68 | | 81.93 |  |  |
|  | | M1 | 10 | 1.03 (0.50-2.15) | | 6 | | 60.00 |  |  |
|  | | TNM stage |  |  | |  | |  | 0.802 |  |
|  | | I+II | 52 | 1.41 (0.73-2.08) | | 41 | | 78.85 |  |  |
|  | | III+IV | 25 | 1.63 (0.55-2.42) | | 18 | | 72.00 |  |  |
| Non-  CRC | CRA | Age | 59.00 (50.00-65.00) | | | | | | | |
|  |  | Gender | 532 |  |  | |  | | 0.619 |  |
|  |  | Male | 338 | 0.84 (0.35-1.40) | 196 | | 57.99 | |  |  |
|  |  | Female | 194 | 0.91 (0.39-1.44) | 113 | | 58.25 | |  |  |
|  | HC | Age | 55.00 (48.00-63.00) | | | | | | |  |
|  |  | Gender | 1099 |  |  | |  | | 0.001 |  |
|  |  | Male | 465 | 0.47 (0.20-1.06) | 185 | | 39.78 | |  |  |
|  |  | Female | 634 | 0.39 (0.15-0.77) | 177 | | 27.92 | |  |  |

^a^ Pathological diagnosis of endoscopic biopsy specimens

^b^ Diagnostic efficiency ITGB4 of the ITGB4 clinical cut-off value (0.70 ng/mL)

^c^ Statistical analysis of ITGB4 concentration within the same group

^d^ Statistical analysis between CRC group and HC group

Supplementary Table 3. The information of metal conjugated antibodies panel

| ***Metal/channels*** | ***Parameters/antibodies*** |
| --- | --- |
| Pd102Di | Barcode |
| Pd104Di | Barcode |
| Pd105Di | Barcode |
| Pd106Di | Barcode |
| Pd108Di | Barcode |
| Pd110Di | Barcode |
| Ce140Di | Beads |
| Pr141Di | EpCam |
| Ce142Di | Beads |
| Nd144Di | IL-4 |
| Nd148Di | GAL |
| Nd150Di | MIP-1 |
| Eu151Di | IL-5 |
| Sm152Di | TNFα |
| Eu153Di | Beads |
| Sm154Di | CD45 |
| Gd156Di | IL-6 |
| Gd158Di | IL-2 |
| Gd160Di | CD14 |
| Dy164Di | IL-17 |
| Ho165Di | Beads |
| Er168Di | IFNg |
| Er170Di | CD3 |
| Yb171Di | GranzymB |
| Yb173Di | ITGB4 |
| Yb174Di | CK8/18 |
| Lu175Di | Perforin |
| Ir191Di | DNA |
| Ir193Di | DNA |
| Pt195Di | Live/Dead |

Supplementary Table 4. The information of 34 potential CRC diagnosis associated proteins

| **Gene Symbol** | **Gene Name** | **Fold change^a^** |
| --- | --- | --- |
| KRT5 | keratin 5 | 1046.7 |
| CST1 | cystatin SN | 496.0 |
| TGM2 | transglutaminase 2 | 296.9 |
| SLC7A5 | solute carrier family 7 member 5 | 162.6 |
| FGF19 | fibroblast growth factor 19 | 151.8 |
| SLC2A3 | solute carrier family 2 member 3 | 122.4 |
| PRF1 | perforin 1 | 116.3 |
| DHRS2 | dehydrogenase/reductase 2 | 103.4 |
| ANO1 | anoctamin 1 | 67.6 |
| CST4 | cystatin S | 52.7 |
| KRT13 | keratin 13 | 49.7 |
| ALDH1A3 | aldehyde dehydrogenase 1 family member A3 | 49.4 |
| PPL | periplakin | 42.0 |
| ITGB4 | integrin subunit beta 4 | 35.2 |
| PSAT1 | phosphoserine aminotransferase 1 | 34.5 |
| SLC1A5 | solute carrier family 1 member 5 | 33.8 |
| INHBB | inhibin beta B subunit | 28.7 |
| LAMA5 | laminin subunit alpha 5 | 24.8 |
| MIA | melanoma inhibitory activity | 22.4 |
| LIF | leukemia inhibitory factor | 20.6 |
| EPS8L2 | EPS8 like 2 | 15.8 |
| TNFRSF6B | TNF receptor superfamily member 6b | 15.5 |
| FKBP4 | FK506 binding protein 4 | 11.8 |
| ITGB5 | integrin subunit beta 5 | 11.5 |
| WNT5A | Wnt family member 5A | 11.0 |
| MDK | midkine | 10.6 |
| ACTN4 | actinin alpha 4 | 9.3 |
| PLEC | plectin | 8.3 |
| CTDSPL | CTD small phosphatase like | 7.4 |
| CSF2 | colony stimulating factor 2 | 7.0 |
| PLOD3 | procollagen-lysine,2-oxoglutarate 5-dioxygenase 3 | 6.7 |
| PLP2 | proteolipid protein 2 | 5.7 |
| COPS6 | COP9 signalosome subunit 6 | 5.2 |
| KRT18 | keratin 18 | 5.2 |

^a^ Fold change was calculated by the results of BIOGPS (http://ds.biogps.org/?dataset=GSE1133&gene=3691, Dataset: GeneAtlas U133A, gcrma)

Supplementary Table 5. Performance of ITGB4 or CEA alone and in combination with eachother for colorectal cancer diagnosis in distinguishing CRC (N=98) from HC (N=1099)

|  | **ITGB4 (cut-off=0.7 ng/mL)** | | **CEA**  **(5 ng/mL)** | **ITGB4 (cut-off=1.6 ng/mL)** | |
| --- | --- | --- | --- | --- | --- |
|  | **ITGB4** | **ITGB4+CEA** |  | **ITGB4** | **ITGB4+CEA** |
| **Ture Positive (N)** | **78** | **87** | **32** | **51** | **70** |
| **False Positive (N)** | **439** | **465** | **47** | **116** | **160** |
| **False Negative (N)** | **20** | **11** | **66** | **47** | **28** |
| **Ture Negative (N)** | **660** | **634** | **1052** | **983** | **939** |
| **Sensitivity** | **79.6%** | **88.8%** | **32.7%** | **52.0%** | **71.4%** |
| **Specificity** | **60.1%** | **57.7%** | **95.7%** | **89.4%** | **85.4%** |
